# Initiation of V(D)J Recombination by Dβ-Associated Recombination Signal Sequences: A Critical Control Point in TCRβ Gene Assembly

**DOI:** 10.1371/journal.pone.0004575

**Published:** 2009-02-24

**Authors:** Don-Marc Franchini, Touati Benoukraf, Sébastien Jaeger, Pierre Ferrier, Dominique Payet-Bornet

**Affiliations:** 1 Centre d'Immunologie de Marseille-Luminy, Université Aix Marseille, Marseille, France; 2 CNRS, UMR6102, Marseille, France; 3 Inserm, U631, Marseille, France; University of Miami, United States of America

## Abstract

T cell receptor (TCR) β gene assembly by V(D)J recombination proceeds via successive Dβ-to-Jβ and Vβ-to-DJβ rearrangements. This two-step process is enforced by a constraint, termed beyond (B)12/23, which prohibits direct Vβ-to-Jβ rearrangements. However the B12/23 restriction does not explain the order of TCRβ assembly for which the regulation remains an unresolved issue. The initiation of V(D)J recombination consists of the introduction of single-strand DNA nicks at recombination signal sequences (RSSs) containing a 12 base-pairs spacer. An RSS containing a 23 base-pairs spacer is then captured to form a 12/23 RSSs synapse leading to coupled DNA cleavage. Herein, we probed RSS nicks at the TCRβ locus and found that nicks were only detectable at Dβ-associated RSSs. This pattern implies that Dβ 12RSS and, unexpectedly, Dβ 23RSS initiate V(D)J recombination and capture their respective Vβ or Jβ RSS partner. Using both *in vitro* and *in vivo* assays, we further demonstrate that the Dβ1 23RSS impedes cleavage at the adjacent Dβ1 12RSS and consequently Vβ-to-Dβ1 rearrangement first requires the Dβ1 23RSS excision. Altogether, our results provide the molecular explanation to the B12/23 constraint and also uncover a ‘Dβ1 23RSS-mediated’ restriction operating beyond chromatin accessibility, which directs Dβ1 ordered rearrangements.

## Introduction

Immunoglobulin (Ig) and T-cell receptor (TCR) genes are assembled from separate variable (V), diversity (D) and joining (J) gene segments via a series of site-specific events of DNA rearrangement, termed V(D)J recombination. This process requires the binding of the lymphocyte-specific recombination activating gene 1 and 2 (RAG1/2) protein complex to recombination signal sequences (RSSs) flanking the rearranging sides of individual V, D and J gene segments [1]. These RSSs consist of conserved heptamer and nonamer sequences, separated by a spacer of 12 or 23 base pairs (bp) of relatively non-conserved DNA. Efficient recombination involves pairs of gene segments flanked by dissimilar 12- and 23RSSs (the 12/23 rule) [2].

**Figure 1:**
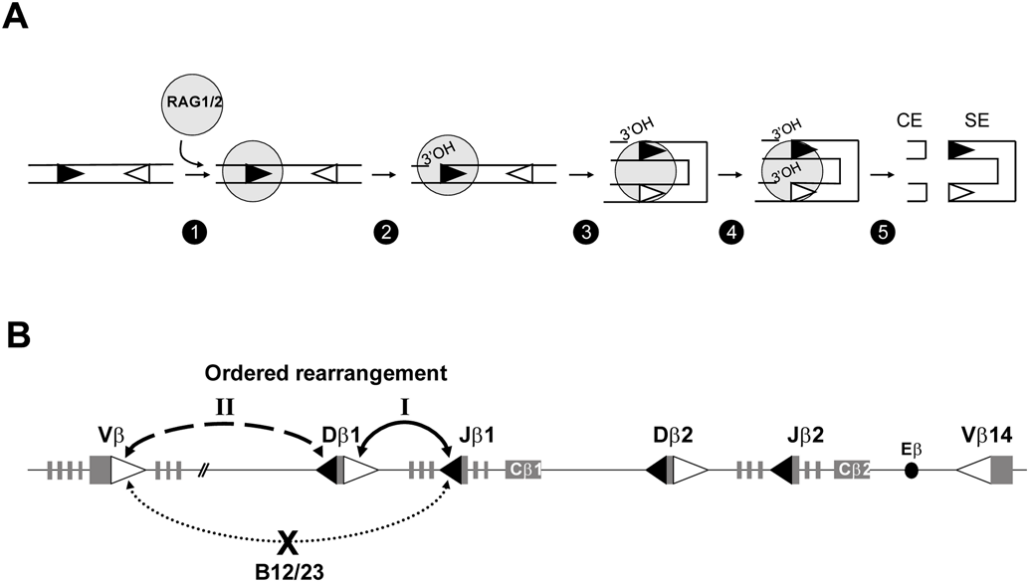
Initial steps of V(D)J recombination and structure of mouse TCRβ locus. (A) According to the capture model initially proposed by Jones and Gellert [8], RAG1/2 complex binds to one RSS (step 1) and then captures the second RSS to form the PC (step 3). Within the PC, pairwise double-strand breakages occur via coupled transesterification reactions, thus leading to the production of SE and CE (step 5). Within this reactions pathway Curry et al. [9] proposed the order of the two nicking reactions; the first one occurs at the initiating RSS (black triangle) (step 2), the second one occurs at the captured RSS (white triangle) (step 4). An alternative model in which the first nick would occur at the captured RSS was considered in the Supplementary Text S1. (B) Schematic depiction of the TCRβ locus. 12- and 23RSSs are represented by black and white triangles, respectively. Gene segments are figured by grey rectangles. TCRβ locus rearrangements are ordered (Dβ-to-Jβ occur before Vβ-to-DJβ). The B12/23 constraint prohibits direct Vβ-to-Jβ rearrangements.

The molecular mechanism of V(D)J recombination has been described in great detail [3]–[5]. Upon binding, the RAG1/2 recombinase introduces a single-strand nick at the border between the RSS heptamers and adjacent coding sequences, thus exposing a 3′-hydroxyl (OH) group on each coding flank. The 3′-OH then attacks the opposite DNA strand in a direct transesterification reaction producing a hairpin-sealed coding end (CE) and blunt phosphorylated signal end (SE). Transesterifications occur simultaneously at complementary RSSs paired within a synaptic or paired complex (PC), a property referred to as coupled cleavage. The processing and joining of CEs and SEs, mediated by DNA repair factors of the nonhomologous end-joining (NHEJ) machinery [6], yield one signal joint and one coding joint as the final products of recombination. The critical event of PC formation likely proceeds via a capture mode in which RAG1/2 complex assembles on one RSS and then captures the second RSS as recombinase-free DNA (**Figure 1A**) [7]–[9].

A tight regulation of V(D)J recombination ensures proper lymphocyte development and eludes lymphoid malignancy-causing chromosomal translocations [3], [5], [10], [11]. Regulated control of V(D)J rearrangement during lymphoid cell ontogeny includes, (*i*) cell lineage specificity (with for example TCR gene rearrangement occurring in T lymphocytes only); (*ii*) developmental specificity (with for example TCRβ gene rearrangement occurring prior to that of TCRα); and, at some loci, (*iii*) allele specificity (to mediate allelic exclusion). By and large, these controls are thought to involve lineage- and developmentally-regulated changes in chromatin structure that precisely modulate the accessibility of individual Ig/TCR gene loci and/or segments, with their associated RSSs, to the unique RAG1/2 recombinase [3], [5], [10].

Beyond the chromatin barrier, individual 12- and 23RSS-flanked gene segments can still display high disparity in recombination frequency, mainly due to nucleotide variations in their RSSs and/or adjacent coding flanks [12]–[14]. In fact, RSS heterogeneity is a major reason for non-random usage in V(D)J recombination. Moreover RSSs can impose significant constraints on antigen receptor gene assembly beyond enforcing the 12/23 rule [15]. Revealed at the TCRβ locus, this B12/23 restriction allows Dβ 12RSSs but not Jβ 12RSSs, to efficiently target Vβ 23RSSs for rearrangement. With unique dependence on the RAG1/2 apparatus and no other lymphoid-specific factors, B12/23 relies on the RSS nucleotides structure and occurs at or prior to coupled cleavage [15]–[24]. However, this phenomenon, which in preserving Dβ gene segment utilization contributes to the optimal generation of a functionally diverse repertoire, remains incompletely understood at the molecular level [22]. Furthermore, while both Vβ-to-Dβ and Dβ-to-Jβ are allowed by B12/23 restriction, an additional level of regulation ensures an ordered V(D)J recombination at the TCRβ locus, with Dβ-to-Jβ joining occurring before Vβ-to-DJβ gene assembly [25] (**Figure 1B**). Although differential chromatin accessibility of TCRβ gene segments may control the rearrangement order, the molecular basis of this process remains however unclear (reviewed in [26]). In this regard, we wondered whether TCRβ RSSs could also organize ordered recombination by orchestrating synaptic complex nucleation in a sequential manner. By investigating RAG1/2-dependent DNA cleavages *in vivo* and *in vitro*, we provide evidence that, at the TCRβ locus, Dβ-flanking 23- and 12RSSs constitute primary anchoring sites for PC formation for D-to-J and V-to-DJ rearrangements respectively. Most importantly, we found that the Dβ1 23RSS also prohibits RAG1/2-mediated nicking at the adjoining 5′Dβ1 12RSS. These data elucidate the mechanism of B12/23 and reveal a role for the Dβ1 23RSS in imposing ordered (‘D-J prior to V-DJ’) rearrangement at the Dβ1 locus.

## Results

### Nicking products preferentially accumulate at Dβ-associated RSSs *in vivo*

**Figure 2:**
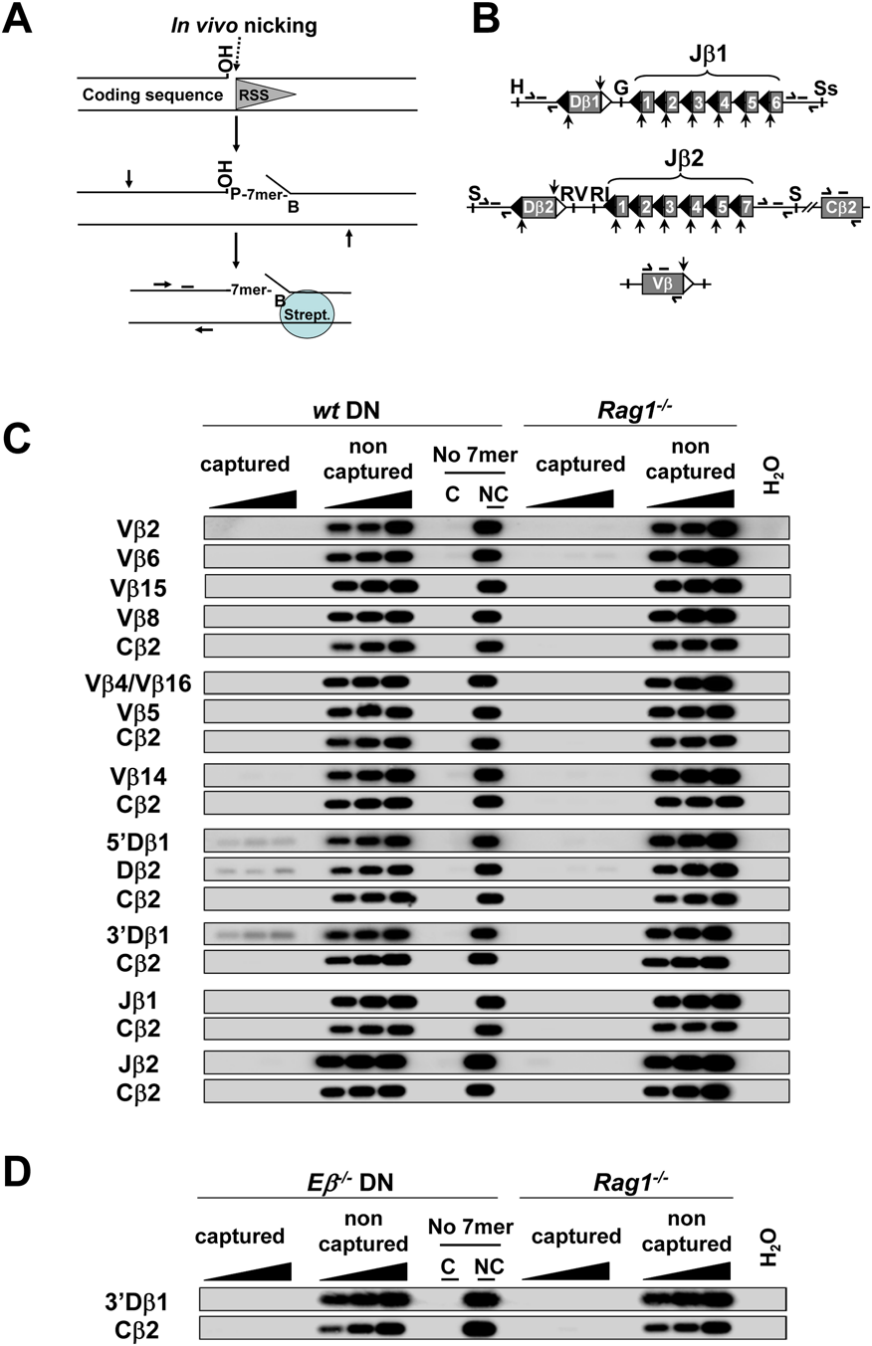
RSS nicks at the TCRβ locus in mouse developing T cells. (A) Strategy to detect RSS nicks *in vivo* using oligo-capture as described by [9]. Vertical and horizontal arrows schematize, respectively, sites for restriction enzyme digestion and primers for PCR amplification. The bar schematizes the hybridization probe used for Southern blot analysis. (B) Schematic view of the TCRβ regions analyzed in this study (not drawn to scale); 12- and 23RSSs are figured by black and white triangles, respectively; the single strand nick positions are indicated by vertical arrows. The locations of the PCR primers and hybridization probes are shown; H (*Hind*III); G (*Bgl*II); Ss (*Sst*I); S (*Sph*I); RV (*Eco*RV); RI (*Eco*RI). (C, D) Autoradiographs of Southern blots of oligo-captured DNAs. Total genomic DNA from WT, RAG1^−/−^ or Eβ^−/−^ DN thymocytes was investigated for single strand nicks at TCRβ RSSs or Cβ2 sequences (used as a negative control). Nicks at Vβ4 and Vβ16 genes were analyzed together; Vβ8 and Vβ5 corresponded to RSSs from three (Vβ8.1, Vβ8.2 and Vβ8.3) and two (Vβ5.1 and Vβ5.2) genes, respectively; single-strand nicks at Dβ2 12- and 23RSSs were analyzed conjointly as these two RSSs possess identical heptamers. Jβ1 and Jβ2 corresponded to all functional Jβ1 and Jβ2 12RSSs, respectively. They were analyzed in one single round using a mixture of specific heptamers followed by PCR amplification of a genomic fragment located at the 3′ end of the Jβ1 (or Jβ2) cluster. PCR reactions were carried out using increasing amounts of template DNA from the bead release (0.5, 1 and 2% of captured DNA) or the flow through (10, 25 and 50 ng of non-captured DNA). Additional controls used 2% of captured (C) and 50 ng of non captured (NC) fractions from genomic DNA treated in parallel except that the biotinylated oligonucleotide was omitted (No 7mer). Estimation of the amount of captured DNA. Assuming that the amount of genomic DNA is 6 pg per cell, the cellular equivalent of 10 ng of genomic DNA is 1650 cells or 3300 alleles. For the less efficient Dβ RSS (5′Dβ1 12RSS) the intensity of the band (when 2% of captured DNA is analyzed) is tenfold lower than the band of non-captured DNA (10 ng of DNA analyzed). Hence, for 2% of captured 5′Dβ1, we estimated that 330 Dβ1 alleles were amplified and that the total amount of captured 5′Dβ1 DNA is around 16500 Dβ1 alleles. We observed that the signal is well detected when approximately 80 copies were analyzed (0.5% of 5′Dβ1 captured DNA). Conversely, nicked Vβ and Jβ RSSs were not detected (even with an input of 10% of captured DNA, not shown), suggesting that there is less than 80 copies of Vβ or Jβ DNA in the PCR tube. If we considered that the efficiency of the oligocapture assay is similar for all DNA targets and that only the amount of nicked DNA varies, we estimated that the amount of nicked Vβ or Jβ RSSs is at least twentyfold lower than the amount of nicked Dβ.

The oligo-capture assay, initially described by Curry et al. [9] (**Figure 2A**), uncovers RAG1/2-mediated nicks generated at a given RSS site(s) in the genome. When applied to the analysis of nicking profiles within the Igκ, IgH and TCRα loci from RAG1/2-expressing cells, this methodology provided evidence that 12RSSs represent initial nicking targets, nucleating synaptic complex formation and the capture of a 23RSS partner [9].

We used the oligo-capture approach to probe RSS nicks associated with rearranging TCRβ gene segments in early developing T lymphocytes. Briefly, genomic DNA from cell-sorted CD4^−^CD8^−^ double-negative (DN) thymocytes of a WT mouse was oligo-captured using heptamer-specific oligonucleotides, T4 DNA ligase and proper restriction enzymes. Next, the digested DNAs were fractionated using streptavidin-conjugated magnetic beads and the captured DNAs tested for the presence of TCRβ sequences of interest using PCR and Southern blotting (**Figure 2 A–B** and see materiel and methods for details). Among all Vβ, Dβ and Jβ RSSs tested, we only detected signal for 5′Dβ1, 3′Dβ1 and Dβ2 captures (**Figure 2C**; nicking at the two neighboring Dβ2 12- and 23 RSSs cannot be distinguished due to the presence of identical heptamers). These signals were above the background level and were specific from WT DN cells. As a negative control, we used genomic DNA from RAG1-deficient (RAG1^−/−^) thymocytes. We also assessed background level from DNA samples treated in parallel but omitting the heptamer oligonucleotide. Finally, each captured DNA at Jβ, Dβ or Vβ gene segments were compared with that at a Cβ2 gene fragment lacking RSS sequences.

According to the previous study suggesting that the 12RSS initiates V(D)J recombination and captures the 23RSS, we expected to observe some nicks at Jβ 12RSS. However, we didn't detect any oligo-captured Jβ1 or Jβ2 DNA. Of note, a greater number of Jβ gene segments cannot explain the difference between the amounts of Dβ versus Jβ capture since we investigated all segments together within each Jβ1 or Jβ2 genomic cluster (see legend to **Figure 2** and **Table S1**). As expected, nicks at Vβ 23RSSs were not detected (**Figure 2C**). Outnumbered targets is also unlikely to account for Vβ *vs.* Dβ differential recovery since when focusing on the Vβ8.1/8.2/8.3 segments (also analyzed together) representing ∼20% of total Vβ rearrangements [27], we still could not detect amplification signals upon using 5 fold more captured DNA (data not shown). We tested the ability of the consensus heptamer CACAGTG (used for the capture of endogenous Vβ2, Vβ6, Vβ8, Vβ15 and Jβ1.1 gene segments) to capture DNA which was previously nicked *in vitro*. The results indicate that the pCACAGTG-biotin heptamer can capture an RSS carrying a RAG1/2-mediated nick (**Figure S1**) and thus does not present any inherent problem. The oligocapture assay appears to be not sensitive enough to detect Vβ or Jβ nicks, mainly two explanations can be considered, either the amount of Vβ or Jβ RSS nicks is underneath the detection threshold or, as discussed below, these nicks exist only transiently.

To verify that RAG1/2 cleavage activity is primarily dependant on RSS accessibility, we used DNA from TCRβ enhancer-deleted (Eβ^−/−^) thymocytes in which Dβ-Jβ clusters display a heterochromatin structure [10], [28]. In contrast to the WT situation, we could not detected any 3′Dβ1 capture (**Figure 2D**), confirming that nicking at the Dβ1 23RSS depends on Eβ-mediated modulation of chromosomal accessibility at this site.

Altogether, our data clearly indicate that rearranging Dβ gene segments *in vivo* contain precisely positioned nicks at their 12- and/or 23RSSs, whereas their potential Vβ and Jβ partners still carry intact complementary RSSs. These profiles argue for a capture mode of PC formation *in vivo* in which Dβ 12- and 23RSSs capture Vβ 23RSS and Jβ 12RSS respectively. The *in vivo* assay failed to detect Vβ or Jβ RSS nicks which likely occur upon formation of the PC (**Figure 1A**, step 4). As discussed by Curry et al., this may signify that nicking at the paired RSS exists only transiently in PCs *in vivo* due to the *quasi*-instant nucleophilic attack in direct transesterification [9]. The oligo-capture assay uncovers RAG1/2-mediated nicks and is not a direct measure of RAG1/2 binding to DNA. Therefore we cannot state about the RAG1/2 binding pattern. Hence we cannot exclude that RAG1/2 initially binds to Jβ or Vβ RSS and that the resulting complex synapses with a Dβ RSS which is next nicked. This alternative scenario is considered in the supplementary textS1.

In conclusion, the *in vivo* nicking pattern of the TCRβ locus strengthens the capture model for synapsis. However, our data suggest that the 12RSS nick leading to the 23RSS capture is not the only order of event; alternatively the initial RAG1/2–mediated cleavage can occur onto a 23RSS such as the Dβ 23RSSs during Dβ-to-Jβ rearrangements. Furthermore, since neither the Vβ 23RSSs nor the Jβ 12RSSs efficiently anchor RAG1/2 cleavage activity, direct Vβ-to-Jβ recombination is prohibited. This anchoring hierarchy represents very likely the molecular basis of the B12/23 restriction at the TCRβ locus.

### B12/23 restriction results from the inefficiency of Vβ 23RSS and Jβ 12RSS to form functional single complex

Previous studies have demonstrated that the B12/23 restriction can be recapitulated *in vitro* with chromatin-free substrates [17]–[19], [24]. Thus, we undertook to use an *in vitro* RAG1/2-mediated DNA cleavage system to validate our proposition that Vβ and Jβ RSSs are captured by Dβ RSSs and therefore cannot recombine together.

As a source of recombinase activity, we used a cellular extract prepared from the D10 cell line [29] after heat-shock induced expression of core RAG1/2 proteins. This extract (hereafter RAG1/2 extract) has been shown to enforce the 12/23 rule *in vitro* [30]. Our various attempts to perform cleavage assays with an *in vitro* system using purified core RAG1/2 and HMGB1 proteins were unsuccessful. This observation is consistent with a previous study in which the Dβ2 23RSS was replaced by the Jκ1 23RSS because the level of recombination of the natural Dβ 23RSS-Jβ 12RSS pair was too low to be properly investigated [14]. The necessity to use crude extracts may suggest that RAG-mediated cleavages on TCRβ RSS-based substrates require, besides RAG1/2 and HMGB1, additional factors. This suggestion is consistent with a recent study indicating that c-Fos would be involved in RAG deposition on Dβ 23RSS [31]. Western blot analysis revealed that our cell-free system supplies the c-Fos protein (not shown).

In addition to the 12- and 23RSSs flanking each Dβ1 and Dβ2 gene segments, we tested the frequently used Jβ1.1- and Jβ2.5 12RSSs [32]; the Vβ2 23RSS, comprised of genuine heptamer and nonamer consensus motifs; and the Vβ14 23RSS, used previously to define and analyze the B12/23 constraint [16], [20]. Sequences of the RSSs analyzed in this study are shown in **Table S2**.

To test our *in vitro* system, we investigated the RAG1/2-mediated DNA coupled cleavage using various pair-wise RSS combinations. As shown in **Figure S2**, this system faithfully reproduced B12/23 restriction and our results are consistent with published data (reviewed in [22]).

**Figure 3:**
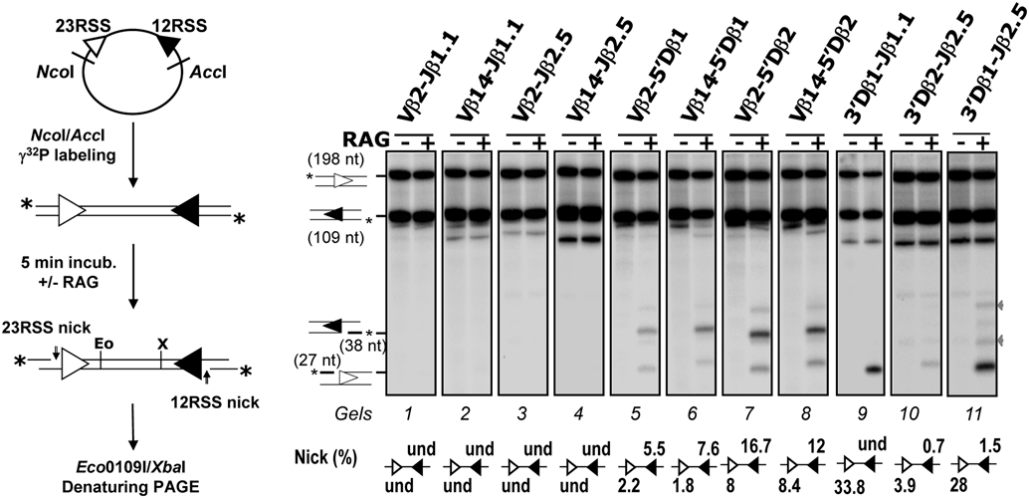
*In vitro* RAG1/2-mediated nicking assays. As described on the left panel, the recombination substrate was first digested with *Nco*I and *Acc*I restriction enzymes and the resulting 12/23 RSS-containing fragment was radio-labeled at the 5′ends (indicated by a star), then incubated for 5 min without (−) or with (+) the RAG1/2 extract. DNA samples were further digested with *Eco*0109I (Eo) and *Xba*I (X) enzymes and separated by denaturing PAGE. (Right) Autoradiographs of nicking assay analysis of the indicated recombination substrates. 12/23 RSS substrates were named according to the gene segments flanking the 23- and 12RSS (see Figure S6 for the construction of recombination substrates). The sizes of the intact 23- and 12RSSs (198 and 109 nt) and of the corresponding nicking products (27 and 38 nt) are indicated. Percentages of 12- and 23RSS single strand nicks (*i.e.*, scanning intensity of individual nicked products *vs.* that of the corresponding original fragments) are shown below the gel image (und: undetected). For some substrates, two additional products of ∼35 and ∼45 nt in length (indicated by grey arrows) were detected in the RAG positive lane, they may correspond to non-hairpin CE DNA breaks (*i.e.*, processed products of RAG1/2-generated hairpins) [46]. All results shown are representative of at least three separate experiments.

Next, we adapted this *in vitro* system to investigate the earliest catalytic phase (RAG1/2-mediated nicking) of V(D)J recombination and especially the aptitude of RSSs to form a functional RAG∶RSS single complex, visualized by the production of single-strand nicks. To do so, the incubation with the RAG1/2 extract was limited to 5 min, and the two 38 and 27 nucleotides (nt) fragments corresponding to respectively RAG1/2-mediated 12- and 23RSS nicks, were monitored (**Figure 3**). When testing Vβ-Jβ substrates, nicking products were not detected (**Figure 3**, gels 1–4). By contrast, we found nicking products from the rearranging Vβ-Dβ substrates, with nicks at the Dβ 12RSSs (38 nt) prevailing over nicks at the Vβ 23RSSs (27 nt) (**Figure 3**, gels 5–8). The detection of higher amounts of the 38 nt fragment complies with our suggestion that Dβ 12RSSs are targeted first for RAG1/2 nicking and PC nucleation. Moreover, we observed that the amount of nicked Vβ 23RSS rose from undetected, for Vβ-Jβ substrates, to ∼2–8% for Vβ-Dβ substrates. The capture model implies that synapsis precedes nicking at the captured RSS (**Figure 1A**, step 3). Therefore, we reasoned that if Vβ 23RSS has to be captured to form the synapse, such capture is dependent on the 12RSS partner. If the 12RSS partner (for instance Jβ 12RSS) cannot initiate the formation of the synapse, Vβ 23RSS would not be nicked, while a 12RSS competent for synapse nucleation would induce Vβ 23RSS nicking. Our observation that nicks are increased at Vβ 23RSSs when associated with Dβ (in comparison with Jβ) 12RSSs thus supports the capture model of Figure 1A and confirms that Dβ 12RSSs represent the platforms of choice for PC nucleation in Vβ/Dβ partnership.

Strikingly, when testing Dβ1-Jβ substrates, we detected large amounts of nicked products at the 23RSS while nicks at the 12RSS were either not detected (3′Dβ1-Jβ1.1 substrate) or quite low (∼1.5% for 3′Dβ1-Jβ2.5 substrates). This outstanding asymmetry is consistent with a model of PC nucleation whereby the RAG1/2 proteins first react with the Dβ1 23RSS before the capture of a free Jβ 12RSS. Nicking profiles of Dβ2-Jβ2 and Dβ1-Jβ substrates are qualitatively similar. However, Dβ1 and Dβ2 23RSS yielded different amount of single strand nicks, respectively ∼30% and 4% of input material (**Figure 3**, gels 9 to 11), implying that the Dβ1 23RSS surpasses the Dβ2 ortholog as a nicking target (hence PC nucleating site) *in vitro*.

Altogether, our *in vitro* data using non chromatinized templates shows that RAG1/2 catalysis preferentially targets the Dβ 12- and 23RSSs, likely nucleating the formation of Dβ/Vβ and Dβ/Jβ PCs, respectively. This conclusion is consistent with the *in vivo* nicking pattern of the TCRβ locus and confirms our proposition that B12/23 restriction results from the inability of Vβ 23RSSs and Jβ 12RSSs to focus the initial RAG1/2 cleavage activity (nicking), leading to a defect of Vβ/Jβ PC formation.

### Dβ1 12RSS nicks are not detected at germline Dβ1 alleles

**Figure 4:**
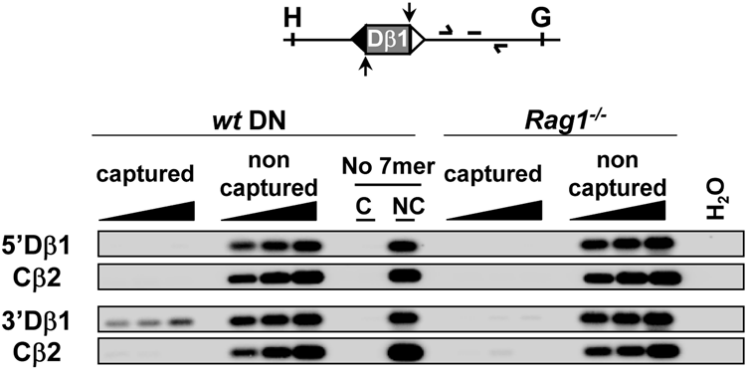
Dβ1 12RSS nicks are not detected at germline Dβ1 locus. Oligocapture assays were performed as described in Figure 2, except that the PCR primers and hybridization probe were specific for sequences in Dβ1-Jβ1 intervening DNA.

Throughout, our data suggest that Dβ-flanking 23- and 12RSSs represent initial RAG1/2-entry sites in Dβ/Jβ and Vβ/Dβ PC formation, respectively. This prompted us to investigate whether the two Dβ-flanking RSSs could be differentially nicked. For this purpose, we used *in vivo* oligo-capture assay at the Dβ1 locus, since (conversely to Dβ2 locus) nicks at Dβ1 12- and 23RSSs can be distinguished due to their divergent heptamers. In our previous oligo-capture assay (**Figure 2**) the 5′Dβ1 and 3′Dβ1 captured DNA were PCR-amplified using primers localized upstream Dβ1 gene segment. For 5′Dβ1 capture, this PCR approach does not differentiate 12RSS nicks at germline Dβ1 and Dβ1Jβ rearranged alleles. Conversely, in the context of 3′Dβ1 capture, this approach detects only 23RSS nicks at germline Dβ1 allele. To detect specifically 12RSS nicks at non rearranged Dβ1 locus we carried out further PCR amplifications from the 5′Dβ1 captured DNAs using primers hybridizing to Dβ1-Jβ1.1 intervening sequences. In this condition, no signal was detected using the 5′Dβ1-captured DNA. When applied to the 3′Dβ1-captured DNA, as expected, this PCR approach (supposed to detect all Dβ1 23RSS nicks, independently to Dβ1 allele configuration) led to the clear detection of the downstream Dβ1 sequence (**Figure 4**). These results clearly show that Dβ1 12RSS nicks are not formed at non-rearranged Dβ1 alleles *in vivo*, while Dβ1 23RSS nicks are produced (**Figure 2**). We deduced that nicking at 5′Dβ1 12RSS occurs after removal of the downstream 23RSS via Dβ-Jβ recombination, only when the allele is in Dβ1Jβ configuration. Here again, the preferential RAG1/2-targeting of 3′Dβ1 23RSS over 5′Dβ1 12RSS would provide an explanation to the ordered rearrangement at the TCRβ locus.

### The Dβ1 23RSS blocks RAG1/2-mediated cleavage at the adjacent Dβ1 12RSS

**Figure 5:**
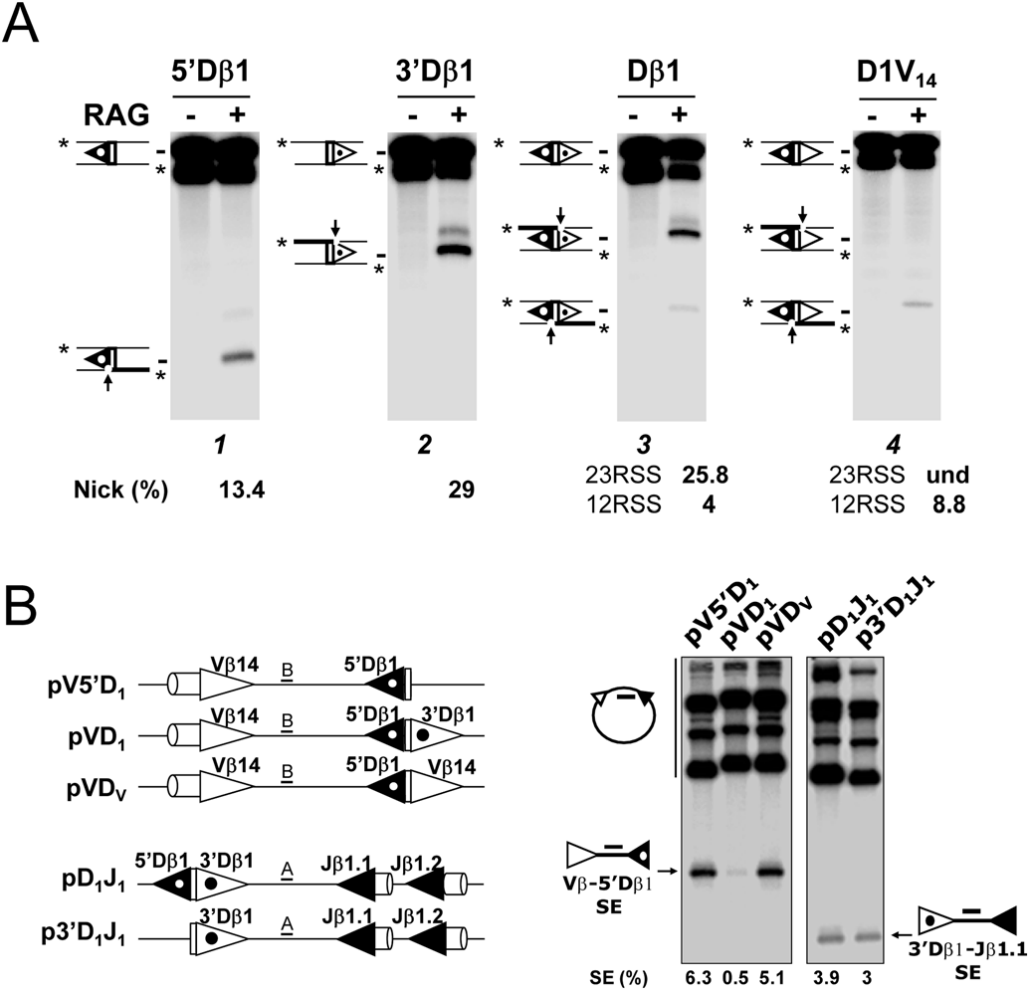
The Dβ1 23RSS impairs RAG1/2-mediated cleavages at the adjacent Dβ1 12RSS *in vitro*. (A) RAG1/2-mediated nicking assays using substrates comprised of the Dβ1 gene segment flanked by various combinations of 5′ and/or 3′ RSSs. The various Dβ-containing fragments were radio-labeled at the 5′ ends and incubated for 5 min without (−) or with (+) the RAG1/2 extract. (B) RAG1/2-mediated coupled cleavage assays of the substrates illustrated on the left. Depending on the substrate, Southern blot analysis used probes A or B, as indicated. (A and B) 12- and 23RSSs are depicted as black and white triangles respectively. Dβ1 RSSs are highlighted by a dot within the triangle. All results shown are representative of at least three separate experiments.

*In vivo*, in the context of an intact chromatin structure, we showed that nicking of the Dβ1 12RSS (and thus initiation of Vβ-to-Dβ1 rearrangement) requires the previous elimination of the Dβ1 23RSS. To test if the inhibition of RAG1/2 cleavage activity on the Dβ1 12RSS is mediated by the neighboring Dβ1 23RSS and not by the chromatin structure, we performed *in vitro* cleavage assays. We first carried out *in vitro* nicking assays using Dβ-based substrates. As previously shown, nicking at a single RSS can occur in presence of Mg^2+^ ions in the buffer [33]. Substrates containing Dβ1 coding sequence flanked by either the Dβ1 12- or 23RSS (5′Dβ1 and 3′Dβ1, respectively) were cleaved in the presence of the RAG1/2 extract to produce the corresponding nicking product (gels 1 and 2, **Figure 5A**). However, a substrate containing the Dβ1 coding sequence flanked by both RSS mostly produced the 23RSS-derived fragment (gel 3) indicating preferential nicking at the Dβ1 23RSS. We observed no such bias towards the 23RSS when using a modified substrate (D1V_14_), in which the Dβ1 23RSS is replaced by the Vβ14 23RSS (gel 4). On the contrary, preferential cleavage fell on the 12RSS. These data therefore suggest a regulatory function unique to the Dβ1 23RSS which, in the germline situation, might anchor RAG1/2 catalytic activity at the expense of the neighboring Dβ1 12RSS.

To further assess the possibility that the Dβ1 23RSS impairs Vβ-to-5′Dβ1 *cis*-rearrangement, we next performed *in vitro* RSS coupled-cleavage assays using various forms of recombination substrates (**Figure 5B**). As demonstrated by the formation of significant amounts of Vβ-5′Dβ1 SE products, coupled cleavage readily occurred using a Vβ14/Dβ1-containing substrate lacking the Dβ1 23RSS (pV5′D_1_) and a related substrate carrying the Vβ14 23RSS at the 3′side of Dβ1 gene segment (pVDv). Conversely, cleavage was severely reduced when using a substrate carrying the Dβ1 23RSS (pVD_1_). Additional experiments demonstrated coupled cleavage within Dβ1/Jβ1-containing substrates whether the Dβ1 12RSS was present or not (pD_1_J_1_ and p3′D_1_J_1_, respectively), arguing that this site has no detrimental effect on PC nucleation involving the downstream 3′Dβ1-/Jβ1-associated RSSs.

Overall, our *in vitro* data using non chromatinized templates demonstrate that RAG1/2 catalysis preferentially targets the Dβ1 23RSS instead of Dβ1 12RSS. Since Dβ1 23RSS mediates the inhibition of the adjacent Dβ1 12RSS nicking, the nucleation of Dβ/Vβ synaptic complex formation is impeded. Thereby, this ‘Dβ1 23RSS-mediated restriction’ provides a potential mechanism to direct ordered rearrangements (‘D-J prior V-DJ’) at the Dβ1 locus.

### Replacement of the Dβ1 23RSS alters the rearrangement order

**Figure 6:**
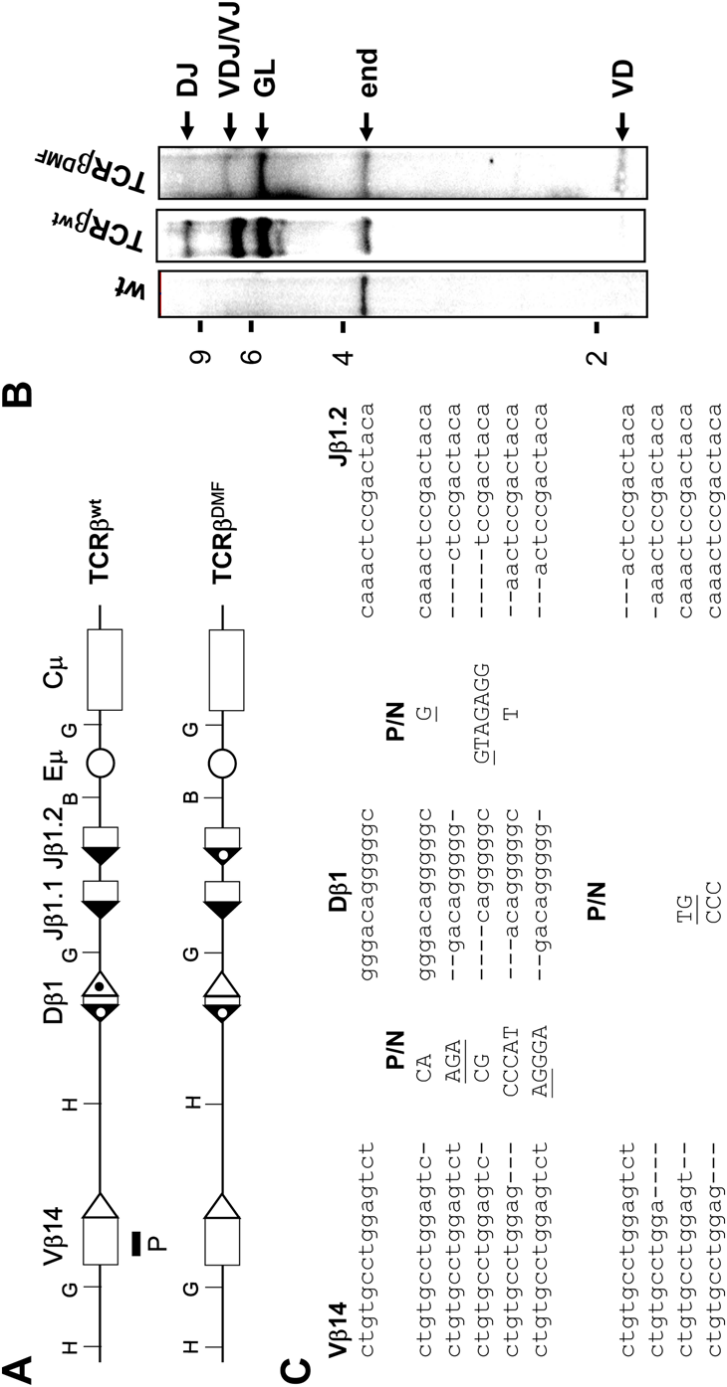
Analysis of TCRβ^DMF^ minilocus rearrangement. (A) TCRβ^wt^ contains germline Vβ14, Dβ1, Jβ1.1 and Jβ1.2 gene segments linked to the IgH intronic enhancer (Eμ) and constant region gene (Cμ). The locations of the Vβ14 probe (P), *Bgl*II (G), *Bam*HI (B) and *Hind*III (H) sites are indicated. 23RSSs (white triangle) and 12RSSs (black triangle) are shown; dotted triangles correspond to the Dβ1 12- and 23RSSs. The TCRβ^DMF^ is similar to the TCRβ^wt^ except that the Dβ1 23RSS and the Jβ1.2 12RSS were replaced with the Vβ14 23RSS and the Dβ1 12RSS, respectively. Miniloci were not drawn to scale. (B) Southern blot analysis of *Bgl*II-digested genomic DNA. DNA was isolated from wild-type thymocytes (wt) and TCRβ^wt^ or TCRβ^DMF^ transgenic thymocytes. The expected size bands from the non rearranged endogenous TCRβ locus (end) and the TCRβ^wt^ or TCRβ^DMF^ minilocus in the non rearranged (GL), DJ, VD and VDJ/VJ configurations are indicated. The 2, 4, 6 and 9 kb markers are shown. (C) Sequences of some VDJ/VJ coding joints from TCRβ^DMF^ rearrangements. Germline coding sequences of Vβ14, Dβ1 and Jβ1.2 are indicated on the top. Nucleotide insertions (P/N) are indicated by capital letters. Presumptive P nucleotides are underlined.

In order to explore further the possibility that an RSS could orchestrate the sequence of VDJ recombination events, we used the transgenic VβDβJβECμ (hereafter TCRβ^wt^) minilocus system. This system has been shown to recapitulate the main features of endogenous TCRβ gene assembly, including B12/23 restriction and ordered TCRβ assembly (i.e. D-J and V-DJ detected in transgenic T cells, but not V-D) [20], [34]. *In vitro* results, using D1V_14_ and pVD_v_ substrates, have shown that Dβ1 12RSS cleavage is not impaired when the Vβ14 23RSS (instead of the Dβ1 23RSS) lies at the 3′side of Dβ1 (**Figure 5**). Since we expected that our *in vitro* system mirrors the *in vivo* situation, we constructed an altered version of the TCRβ^wt^ minilocus in which we replaced the Dβ1 23RSS by the Vβ14 23RSS and the Jβ1.2 12RSS by the Dβ1 12RSS, this yielded the TCRβ^DMF^ minilocus (**Figure 6A**). Theoretically, in this configuration the various VJ1.2, DJ1.2, VD or VDJ1.2 rearrangements are possible since they all comply both with the B12/23 and ‘Dβ1 23RSS-mediated’ restrictions. We generated the TCRβ^DMF^ transgenic mice and then analyzed the genomic DNA isolated from thymocytes by Southern blotting using *Bgl*II restriction enzyme and a probe that spans the Vβ14 gene segment (**Figure 6**). As previously published [20], [34], we observed that the TCRβ^wt^ minilocus undergoes DJ and VDJ rearrangements, but not VD rearrangement. By contrast and as anticipated, VDJ/VJ and VD rearrangements were readily found within the TCRβ^DMF^ minilocus, indicating that the rearrangement order of TCRβ^DMF^ is altered compared to endogenous TCRβ locus or TCRβ^wt^ minilocus (**Figure 6B**). VDJ/VJ joints specific for the TCRβ^DMF^ transgene were analyzed by PCR and some of them were cloned and sequenced (**Figure 6C**). We did not detect any VDJ1.1/VJ1.1 rearrangement in agreement with the B12/23 restriction. Only the Jβ1.2 segment flanked with the Dβ1 12RSS was used either for direct VJ rearrangements (∼40%) or, for VDJ rearrangements (∼50%) (the remaining 10% could not be clearly assigned to either VDJ or VJ joints). This result reproduced previous data indicating that the substitution via knock-in of the Jβ1.2 12RSS by the Dβ1 12RSS results in direct Vβ-Jβ1.2 rearrangements [15]. As DJ joints were not detected by Southern blotting, we deduced that the stepwise order of VDJ assembly for the TCRβ^DMF^ is mainly V-to-D rearrangement, followed by VD-to-J rearrangement. As such, RSS can interfere with the sequential steps of TCRβ gene assembly and the Dβ1 23RSS is crucial for the proper ordered ‘D-J prior V-DJ’ rearrangement. This conclusion is consistent with previous results showing that the mutation of the Dβ1 23RSS leads to the formation of V-D joints [20]; however in this study, the Dβ1 23RSS mutation prevents Dβ-to-Jβ rearrangements consequently no VDJ joints were formed. Thus, an alternative scenario would be that V-D rearrangements occur because the D-J rearrangements are inefficient. In TCRβ^DMF^ transgenic mice the Dβ1 23RSS is replaced by the functional Vβ14 23RSS and as expected we detect some VDJ joints. Moreover, *in vitro* coupled cleavage assays using transgene-based substrates showed that D-J coupled cleavage is not particularly slowed down with pTCRβ^DMF^ substrate compared to pTCRβ^wt^ substrate. On the other hand, with pTCRβ^DMF^ substrate, the V-D coupled cleavage is more efficient than D-J cleavage (**Figure S3**). Therefore this data support our initial scenario; the formation of VD joints in TCRβ^DMF^ minilocus likely results from the inability of the Vβ14 23RSS to restrain the V-D cleavage but not from a defect in D-J cleavage.

Altogether our *in vivo* and *in vitro* data converge towards a model in which the Dβ1 23RSS not only represents a preferential target for RAG1/2 nicking at germline Dβ1 alleles but also prohibits nicks at the adjacent 12RSS, unless removed via 3′ rearrangement. Consistent findings in both *in vitro* and *in vivo* assays strongly suggest that these properties do not rely on a function of the non-core domains of the RAG1/2 or on the selective tuning of chromosomal accessibility on both sides of the Dβ1 segment.

## Discussion

This study shows that TCRβ RSSs, regardless of their structural (12/23) type, display broad disparities in their overall ability to undertake the first catalytic step of V(D)J recombination, RAG1/2-mediated nicking. Within the limits of sensitivity of single strand nick assays, these range from a relatively high potential (Dβ1 23RSS) to lower aptitude (Dβ1 12RSS, Dβ2 12- and 23RSSs) to near ineffectiveness (Vβ 23-, Jβ 12RSSs). The proficiency of the Dβ1 23RSS to undergo RAG1/2-mediated nicking activity is coupled with an inhibition of that at the 5′ adjoining 12RSS. These data have a number of implications for the biology of V(D)J recombination and the control of TCRβ gene assembly. Notably, the emerging picture that nicks preferentially accumulate at Dβ segments strengthens the model that recombination synapsis proceeds via the capture of a free RSS by a RAG1/2-loaded partner [7]–[9]. However, the nucleating site is not necessarily the 12RSS; at the Dβ-Jβ clusters, the Dβ 23RSSs assume this function. At the TCRβ locus, the pattern of nucleating and captured RSS provides an explanation for the B12/23 restriction and reveals how the capture mechanism for PC formation contributes to V(D)J recombination regulation.

We observed an ineffective RAG1/2-mediated nicking of Vβ/Jβ substrates *in vitro*, with complete absence at the endogenous TCRβ locus in WT DN cells. These data strongly argue that one aspect of the B12/23 constraint results from the inability of Vβ 23- and, especially, Jβ 12RSSs to initiate PC assembly, and therefore to form a synapse together. Our data do not establish where RAG1/2 proteins bind; therefore they don't discriminate between two possible hypotheses to explain the scarce nicking at Vβ and Jβ RSSs: these RSSs are poor substrates for either RAG binding or for the RAG nicking reaction *per se*. The first hypothesis is not supported by previous EMSA studies showing that RAG binding to Dβ1 12RSS and to Jβ 12RSSs was equivalent [18], [24]. Moreover, it was proposed that the scarce nicking of Jβ 12RSSs (compared to Dβ 12RSS) results from a slow nicking rate [24]. We note that these EMSA were performed with purified RAG1/2 in Ca^2+^ buffer, thus it cannot be excluded that DNA binding properties of RAG1/2 proteins in Ca^2+^ and Mg^2+^ buffers differ slightly. Also, if we considered that some additional proteins could be involved in RAG binding to RSS [31], the DNA binding properties of RAG1/2 may well vary depending on the system used (purified RAG or cell-free system). Certainly, at one stage of V(D)J recombination Vβ and Jβ are bound and nicked by RAG1/2. Thus, we suggest that during the PC formation the RAG1/2-loaded Dβ 12- or 23RSS locks the RAG1/2 multimers in a conformation [8] favoring either binding or nicking reaction at the captured (Vβ or Jβ) RSS (see Supplementary Text S1).

A previous study has stressed the usual proficiency of 12RSSs to capture their 23RSS partner [9]. This ‘12RSS anchoring model’ is challenged by our suggestion that Jβ 12RSSs are captured by Dβ 23RSSs. We attempted to understand this atypical situation by analyzing DNA sequences. This analysis showed that Jβ RSSs are heterogeneous within each cluster, only few nt are conserved (**Figure S3A)**. Notably, Jβ 12RSS nonamers tend to deviate strongly from the consensus hallmark (more significantly at the Jβ1 cluster). As previously proposed RAG∶RSS complexes may contain two types of interactions: ‘digital’ which involve critical nt residues absolutely required for RSS function and ‘analog’ (or ‘multiplicative’) which involve non critical nt residues that modulate the activity of the RAG∶RSS complex [14]. Probably, Jβ RSSs contain the critical nt (which are well-conserved, for instance d(TGTG) at the 3′end of the heptamer) but do not possess nt residues required for optimal analog contact, thus explaining the atypical inefficiency of Jβ 12RSSs to form functional single complex. It is tempting to speculate that due to such suboptimal function, Jβ RSS sequences may have been selected in order to maintain the B12/23 constraint (*i.e.*, avoid PC nucleation at Jβ RSSs). In contrast heptamers and nonamers of Dβ RSSs are close to the consensus sequences. Additionally, Dβ RSSs display high conservation across distant species (**Figure S4B**). Especially the spacer/heptamer and the spacer/nonamer boundaries are well conserved in Dβ 23RSSs. We performed further *in vitro* cleavage analysis using Dβ1 23RSS carrying mutations in the spacer. The results showed that some mutations in the spacer/heptamer boundary (which comprises a putative d(TGATTCA) AP-1 binding site), affect both nicking and coupled cleavages of 3′Dβ1-Jβ1.1 substrates and also partially abolish the ‘Dβ1 23RSS-mediated restriction’ (**Figure S5**). These results are consistent with the suggestion that the AP-1 site may be crucial for Dβ 23RSS function [31].

The coding sequence affects V(D)J recombination, thus if the heptamer is flanked by a “bad” coding sequence (such as T or A stretch) the recombination efficiency may decrease [13], [35], [36]. Also, it has been demonstrated that d(TTT) coding flank slows down nicking rate but does not interfere with RAG binding [37]. Dβ RSSs are flanked by “good” coding sequences which could account for their higher efficacy to focus RAG-mediated cleavage, compared to Jβ or Vβ RSSs [17]. However this could not explain why Dβ1 23RSS is more efficiently nicked than the Dβ2 23RSS since Dβ1 and Dβ2 RSSs possess identical coding flanks. Thus, the difference in performance of these two RSSs lies in their sequence variation that could be further investigated by RSS mutagenesis. Despite this difference, the coupled cleavages of Dβ1-Jβ1 and Dβ2-Jβ2 substrates are similar and are both weak compared to Dβ1-Jβ2 substrates, suggesting that Jβ2 RSSs are better partners than Jβ1 RSSs (**Figure S2** gels 13 to 18); Jβ2 RSSs may counterbalance the low efficiency of Dβ2 23RSS to focus RAG activity whereas Jβ1 RSSs (likely because of an unfavorable nonamer) may restrain Dβ1 23RSS performance to assemble a functional PC. Thus the likely efficiency of coupled cleavage of a given RSS pair would first depend, on the proficiency of the nucleating RSS to focus RAG activity (such as Dβ1 23RSS>Dβ2 23 RSS and Dβ2 12RSS>Dβ1 12RSS) and then for some RSS pairs, on the aptitude of the captured RSS to be bound by the RAG complex and to possibly undertake the nicking reaction. Furthermore, besides the individual features of the RSS, we should also consider the possibility that depending on the type of nucleating site (12- or 23RSS), the PC assembly could slightly differ which may, to some extent, account for the pair-wise modulation of RAG-mediated cleavages. Indeed, Jones and Gellert have previously pointed out that initial binding onto a 12RSS leads to a more faithful adherence to the 12/23 rule and in explaining this observation, they proposed that the RAG1/2 multimers could be differentially locked depending on the initial binding RSS [8].

Our genome is scattered with sequences akin to RSS (the so-called cryptic RSS), but surprisingly, these cryptic RSSs are rarely mis-targeted by the recombinase (reviewed in [38]). In addition to possessing the critical nucleotides required for RSS function and to be accessible at the time of RAG1/2 expression, the cryptic RSS must find a suitable RSS partner in order to recombine. Such pair-wise modulation of the RAG1/2-mediated coupled-cleavage represents an additional constraint that safeguards the genome against illegitimate recombinations. Indeed, as shown in this study, amongst the TCRβ RSSs tested, only the four Dβ-associated RSSs are competent for the initiation of V(D)J recombination. We hypothesize that most of the cryptic RSS may belong to the captured RSS category, and therefore a productive reaction with RAG1/2 would rely on their RSS partner. Up to now, the fully-characterized V(D)J-mediated translocations resulting from a targeting mistake of the recombinase involving the TCRβ and various oncogenes (Lck, Tal2 or Lmo2) occur between the Dβ1 23RSS and cryptic 12RSS [38]. This observation complies with our scenario; the functional single complex RAG∶Dβ1 23RSS could capture a cryptic 12RSS which may well be (as Jβ1 12RSS) suboptimal, leading to translocation.

According to RAG1/2-mediated cleavage analysis, the Dβ1 23RSS blocks concurrent processing of the *cis*-linked 5′ 12RSS and consequently is likely to be essential for the proper ‘D-J prior V-DJ’ rearrangement order at the TCRβ locus. Our data strongly supports the model in which removal of Dβ1 23RSS through Dβ1-to-Jβ rearrangement is an essential step to eliminate the impediment to Vβ-to-Dβ1 rearrangement. Consistent with this model, if the Dβ1 23RSS is replaced by the functional Vβ14 23RSS (**Figure 6**) or a mutated Dβ1 23RSS [20] VDβ1 joints are then detected. Our data indicate that the Dβ1 23RSS (compared to all other Dβ RSSs) focuses RAG1/2 activity with a greater effectiveness and likely this mediates the inhibiting role of Dβ1 23RSS on the Dβ1 12RSS nicking. Footprinting analysis have shown that in the single RSS∶RAG complex few nt adjacent to the heptamer are protected by RAG1/2, however a much larger region in the coding sequence, at least 12 bp, is protected in the synaptic complex [39]. Therefore as the Dβ1 coding sequence is only 12 bp long, it seems consistent that the RAG∶Dβ1 23RSS complex sterically hinders the formation of a PC involving the Dβ1 12-RSS. Nevertheless, further molecular studies are necessary to clearly define the mechanics of this Dβ1 23RSS-mediated restriction.

Similarly to other DNA transactions, V(D)J recombination is prominently regulated by chromatin structure and modifications [10]. In this context, recent reports showed that RAG2 interacts with histone H3 hypermethylated at lysine 4, an epigenetic mark usually associated with active chromatin [40], [41]. In addition to help RAG1/2 to target loci poised to undergo rearrangement, the authors proposed that this interaction, through allosteric activation of the recombinase, is directly involved in V(D)J recombination reaction. Concerning the TCRβ locus, an increasing body of evidence argues in favor of at least two types of *cis*-acting regulatory elements, the transcriptional enhancer (Eβ) and the germline promoter pDβ1, controlling the initiation of V(D)J recombination [10], [26]. Eβ alone supports chromatin opening along the Dβ-Jβ clusters while an interaction with pDβ1 converts the Dβ1 segment into an accessible site. As shown in **Figure 2D**, the Dβ1 23RSS is not nicked in the Eβ^−/−^ thymocytes confirming that RSS accessibility is a prerequisite for RAG1/2 cleavage activity. Therefore chromatin structure and epigenetic marks, by modulating appropriately RSS accessibility (or inaccessibility) of the various TCRβ gene segments, could be sufficient for a tight regulation of V(D)J recombination. Nonetheless, mechanisms distinct from RSS accessibility exist to ensure B12/23 restriction [22], allelic exclusion [42] and, as shown herein, ordered rearrangement. What is the purpose of such additional regulation mechanisms? As a minimum, they may represent security systems that guarantee proper V(D)J recombination in cases where RSSs are untimely accessible. However a previous report demonstrated that, within CD4^+^CD8^+^ T-cells undergoing V(D)J recombination, Vβ gene segments upstream of a functional VDJβ1 rearrangement are maintained in an active chromatin environment but were still restricted from further rearrangement despite the proximity of Dβ2 gene [43]. This study highlights the possibility that, during normal T lymphocytes development, Vβ, Dβ and Jβ RSSs can be concomitantly accessible; this would therefore justify the existence and preservation of regulation systems operating beyond chromatin accessibility.

## Materials and Methods

### Cells and mice

The D10 cell line [29] was provided by Dr. D.G. Schatz (Yale University School of Medicine, New Haven, CT). Cells were cultured in DMEM supplemented with 10% FCS, 100 U/mL penicillin, 100 µg/ml streptomycin, 50 µM 2-mercaptoethanol; and incubated at 37°C in a humidified chamber containing 5% CO2. C57BL/6J wild-type (WT), RAG1-deficient (RAG1^−/−^) [44], Eβ-deleted (Eβ^−/−^) [45], TCRβ^WT^ [34] and TCRβ^DMF^ mice were housed under specific pathogen-free conditions, and handled in accordance with French and European directives.

### Isolation of CD4^−^CD8^−^ double-negative (DN) thymocytes and DNA purification

Total thymocytes were incubated 1 h at 37°C in the presence of rabbit complement (Low-Tox, Cederlane) and rat IgM anti-mouse-CD4 (RL172.4) and -CD8α (3.155) antibodies. Living cells were collected on a ficoll gradiant (Ficoll-Paque Plus, GE-Healthcare). Cell preparations were >95% DN as determined by flow cytometric analysis. Genomic DNA from purified DN WT, RAG1^−/−^ or Eβ^−/−^ thymocytes was prepared as previously described [28].

### Oligo-capture assays

Analysis of single strand nicking products by oligo-capture assays was performed according to [9], using genomic DNA from DN thymocytes, 5′ phosphorylated, 3′ biotinylated oligonucleotides specific to RSS heptamers within the TCRβ locus, and appropriate restriction enzymes (**Table S1**). Detection of the oligo-captured DNA fragment(s) was carried out by PCR. Briefly, PCR reactions (25 µl in 1× PCR buffer; 3 min at 94°C, followed by 28 cycles of 30 sec/94°C, 60 sec/60°C, 30 sec/72°C, and 7 min at 72°C) contained increasing amounts of either the captured (0.5%, 1% and 2%) or non-captured (10 ng, 25 ng and 50 ng) DNA, specific primers (5 pmol each), 0.2 mM dNTP, 2.5 mM MgCl_2_ and 1 U *Taq* DNA polymerase (Invitrogen). Amplified DNAs were separated through a 1% agarose gel, transferred onto a Biodyne B membrane, and hybridized using a 5′ end ^32^P radio-labeled specific probe (sequences of PCR primers and hybridization probes listed in **Table S3**).

### Plasmid constructs

Substrates for DNA cleavage were constructed using PCR amplified fragments from various genomic DNA regions within the mouse TCRβ locus and standard molecular cloning procedures. PCR amplifications (30 sec/94°C; 30 sec/59°C; 45 sec/68°C; 32 cycles, with a final amplification step at 68°C for 7 min) were performed using Platinum® Taq DNA Polymerase High Fidelity (Invitrogen) and appropriate oligonucleotide primers (**Table S4**). PCR products were purified following electrophoresis through a 1% agarose gel and subcloned into the pGEMT-easy or pGEM-7Zf vectors (Promega). In all constructs, RSSs and adjacent flanking sequences were checked by DNA sequencing (MWG Biotech). In total, three groups of substrates were used. The first two groups comprised DNA plasmids that were derived from either a construct containing a 657 bp Dβ1-Jβ1.1 insert (group I; **Figure S6**) or a construct containing a 580 bp Dβ1 overlapping insert (group II; **Figure S7**). A third group comprised four DNA fragments (5′Dβ1, 3′Dβ1, Dβ1, and D1V_14_), individually produced by PCR amplification using a plasmid from group II as template and oligonucleotide primers #181 and #318 (respectively, templates p5′Dβ1, p3′Dβ1, pDβ1 and pDv).

### Protein extracts

The RAG1/2-containing extract was prepared from heat-shocked D10 cells according to a published protocol [29]. Protein contents were determined using the Bio-Rad Protein Assay (Bio-Rad).

### RAG1/2-mediated DNA cleavage *in vitro* assays

RAG1/2-mediated coupled cleavage was performed for 3 h at 30°C in a final volume of 25 µL of cleavage reaction buffer (50 mM Hepes-KOH pH 7.5, 73 mM KCl, 2 mM NaCl, 10 mM MgCl_2_, 1 mM DTT) supplemented with the RAG1/2 extract (20–30 µg), 1.5 mM rATP, and proper recombination substrate (0.3 pmol). To increase cleavage efficiency, 6 µg of a nuclear extract prepared from mouse WT thymocytes were also added. Negative controls were carried out using similar conditions without addition of the RAG1/2 extract. After phenol extraction and ethanol precipitation, the DNA samples were electrophoresed through a 1% agarose gel and analyzed by Southern blot using a Biodyne B transfer membrane (Pall Corporation). Membranes were hybridized with TCRβ-specific, radio-labeled probes A (5′-GAGAAGAGTAGAGGACTGTGGGCCTTGG-3′) or B (5′-GACTTGAATCATGTTGTTTTCC-3′). For RAG1/2-mediated nicking assays, the substrate was first digested by restriction enzymes *Acc*I and *Nco*I. The resulting 700 bp fragment was gel purified (Wizard SV Gel and PCR Clean-Up System, Promega) and radio-labeled at 5′ ends using T4 polynucleotide kinase (Invitrogen) and γ^32^P-[ATP] (GE-Healthcare). The labeled substrate (∼0.1 pmol) was used for DNA cleavage as described above, except that incubation was for 5 min. The DNA samples were then deproteinized and further digested by restriction enzymes *Eco*O109I/*Xba*I (that cut within RSS-intervening sequences) to ensure proper quantification of nicked *vs.* intact RSSs. Formamide loading buffer was added to the digests and samples were heated at 95°C then separated by 15% polyacrylamide gel electrophoresis (PAGE) under denaturing conditions (7 M urea). Nicking assays of the 5′Dβ1, 3′Dβ1, Dβ1 or D1V_14_ amplified products used similar conditions except that the deproteinized samples were electrophoresed directly without further restriction enzyme treatment.

### TCRβ^DMF^ minilocus

To generate the TCRβ^DMF^ minilocus, the Dβ1 23RSS and Jβ1.2 12RSS of the TCRβ^wt^ minilocus described in [34] were replaced by the Vβ14 23RSS and Dβ1 12RSS respectively. Briefly, the TCRβ^wt^ *Hind*III/*Bam*HI fragment containing the germline Dβ1, Jβ1.1 and Jβ1.2 gene segments was first subcloned in pGEMT-7zf, thereby generating pTgDJ. The Dβ1 23RSS mutation was introduced by replacing the pTgDJ *Eco*O109I fragment by the one from the pD_V_ substrate, thus generating pTgD_V_J. The 5′Jβ1.2 12RSS mutation was introduced by a two-steps PCR approach. First, pTgD_V_J was amplified using primers 213/208 and 207/214 to generate the fg1 and fg2 fragments, respectively. Then, a second PCR using fg1, fg2 and 213/214 primers was performed to produce the Jβ1.2 mutated fragment. This latter PCR product was digested with *Eco*RV/*Bam*HI and subcloned into *Eco*RV/*Bam*HI-digested pTgD_V_J construct to produce the pTgD_V_J2_5D_ vector. Finally the *Hind*III/*Bam*HI fragment of pTgD_V_J2_5D_ was inserted into the *Hind*III/*Bam*HI-digested TCRβ^wt^ to produce the TCRβ^DMF^ minilocus. Microinjection of TCRβ^DMF^ into fertilized eggs, production of transgenic mice lines and analysis by southern blot of the rearrangement specific to the minilocus were conducted as previously described [34].

## Supporting Information

Figure S1 Oligocapture mediated by p-CACAGTG-biotin.
(0.06 MB PDF)

Figure S2 Pairwise modulation of RAG1/2-mediated coupled cleavage.
(0.20 MB PDF)

Figure S3 RAG1/2-mediated coupled cleavage of pTCRβWT and pTCRβDMF substrates;
(0.12 MB PDF)

Figure S4 Phylogenetic profiles of Dβ and Jβ RSSs
(0.15 MB PDF)

Figure S5 Effect of Dβ1 23RSS spacer mutations on RAG1/2-mediated cleavages
(0.16 MB PDF)

Figure S6 Cloning strategy to construct recombination substrates
(0.02 MB PDF)

Figure S7 Cloning strategy to construct additional recombination substrates
(0.03 MB PDF)

Table S1 5′-phosphorylated 3′-biotinylated oligonucleotides (7-mers) and restriction enzymes used in the oligo-capture assays to displace and ligate the nicked strand and to restrict the genomic DNA before purification on streptavidin-conjugated magnetic beads.
(0.05 MB PDF)

Table S2 DNA sequences of the RSSs (plus the three proximal nucleotides from coding flanks) used in this study.
(0.01 MB PDF)

Table S3 Oligonucleotide primers and hybridization probes used in the oligo-capture assays for PCR amplification and Southern blotting identification of the captured DNAs
(0.05 MB PDF)

Table S4 Oligonucleotides used in the construction of the various DNA cleavage substrates analyzed in this study.
(0.03 MB PDF)

Text S1 Consideration of an Alternative capture model.
(0.07 MB PDF)
