# Supplementary material for: Initiation of V(D)J Recombination by Dβ-Associated Recombination Signal Sequences: A Critical Control Point in TCRβ Gene Assembly
Source: PLoS One. 2009 Feb 24;4(2):e4575. doi: 10.1371/journal.pone.0004575 (PMC2642999; doi:10.1371/journal.pone.0004575)
Supplement: Figure S1 — Oligocapture mediated by p-CACAGTG-biotin. (0.06 MB PDF) [file pone.0004575.s001.pdf]

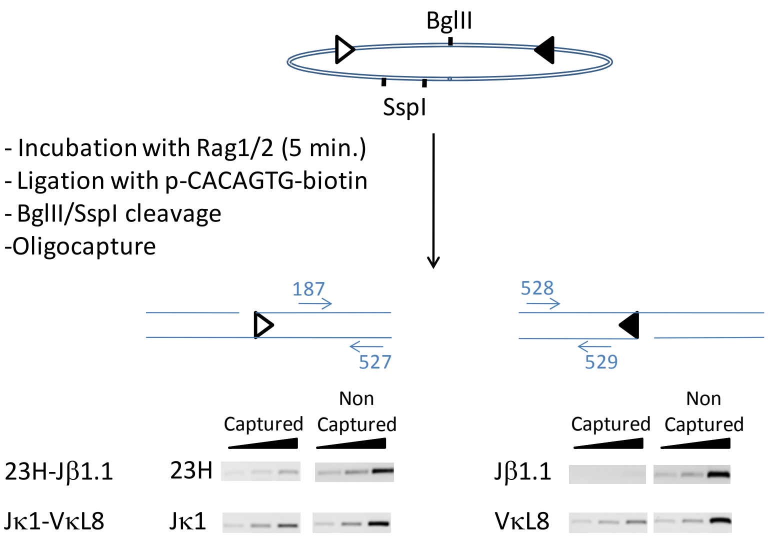

Figure S1: Oligocapture mediated by p-CACAGTG-biotin.

Nicks were introduced in 12/23 RSS substrates using the *in vitro* RAG1/2-mediated cleavage assay. The 23- and 12-RSS are represented by white and black triangles respectively. After 5 min incubation with RAG1/2, the samples were deproteinized and the substrates (25 ng) were incubated with 1.5 pmol of pCACAGTG-biotin heptamer and 5 units of T4 DNA ligase. Following ligation, the DNA was cleaved with BglII and SspI restriction enzymes and then oligocaptured as described in the Material and Method section. Two 12/23 RSS substrates were used: 23H-Jβ1.1 and Jκ1-VκL8. The 23H RSS is identical to 3'Dβ1 23RSS except a point mutation converting the 3'Dβ1 23RSS heptamer d(CACGGTG) into the consensus heptamer d(CACAGTG). The captured and non-captured DNA were analyzed by PCR. The 23RSS and 12RSS-containing fragments were respectively detected using the 187/527 and 528/529 primers. For PCR, we used increasing amount of either captured DNA (0.5%, 1% and 2%) or non-captured DNA (0.005%, 0.01% and 0,05%). Amplified DNA were separated through a 1% agarose gel and then visualized using ultraviolet light. Within the captured fraction, the 23H RSS (but not Jβ1.1 12RSS) containing fragment was easily detect by PCR. For Jκ1-VκL8 substrates both Jκ1 and VκL8 RSS were oligocaptured. Altogether these results are consistent with the *in vitro* nicking profiles indicating that, when testing 23H-Jβ1.1 substrates, nicks are detected only at the 23RSS whereas nicked products were detected at both RSS with Jκ1-VκL8 substrate (not shown).

.
