# Supplementary material for: Initiation of V(D)J Recombination by Dβ-Associated Recombination Signal Sequences: A Critical Control Point in TCRβ Gene Assembly
Source: PLoS One. 2009 Feb 24;4(2):e4575. doi: 10.1371/journal.pone.0004575 (PMC2642999; doi:10.1371/journal.pone.0004575)
Supplement: Figure S2 — Pairwise modulation of RAG1/2-mediated coupled cleavage. (0.20 MB PDF) [file pone.0004575.s002.pdf]

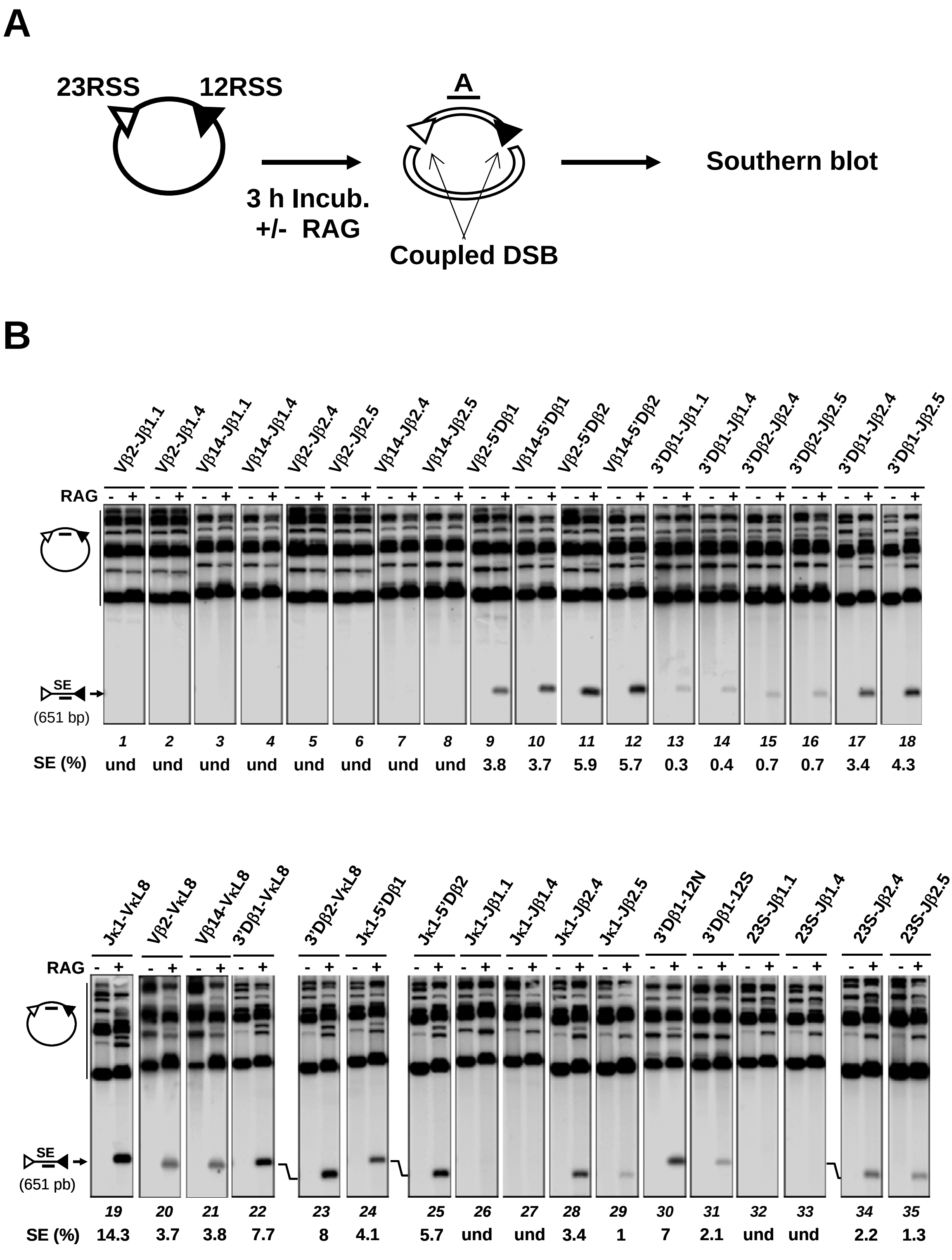

Figure S2. Pairwise modulation of RAG1/2-mediated coupled cleavage.

(**A**) *In vitro* DNA coupled-cleavage assay. As schematized on the left, the 23- and 12RSS-containing substrates were incubated for 3 h without (-) or with (+) the RAG1/2 extract. The cleaved DNA was then separated by gel electrophoresis and the signal end fragment (SE, 651 bp) was revealed by southern blotting. (**B**) Autoradiographic images from coupled-cleavage analysis of the indicated recombination. 12/23 RSS substrates were named according to the

gene segments flanking the 23- and 12RSS (see Figure S6 and Table S2 for substrates construction and DNA sequences of RSS). In the top panel (gels 1 to 18) DNA substrates contain a 12/23 RSS pair from TCRβ gene segments. In agreement with the B12/23 constraint, we did not detect any SE product when using Vβ-Jβ substrates (gels 1-8) while we readily detected coupled cleavage with Vβ-5'Dβ substrates (gels 9-12) and with 3'Dβ–Jβ substrates (gels 13-18). We remarked the variations in the amount of SE products when testing intra- (3'Dβ1-Jβ1 or 3'Dβ2-Jβ2 substrates) *vs*. inter- (3'Dβ1-Jβ2 substrates) locus combinations. In the bottom panel (gels 19 to 35) we exploited the consensual VκL8 12- and Jκ1 23RSSs to decipher the sequences responsible for the B12/23 restriction. We found that each one of the Vβ 23RSSs, 3'Dβ 23RSSs and 5'Dβ 12RSSs interacted efficiently with the corresponding κ partner yielding significant levels (3.7% to 8%) of SE products (gels 20-25). Notably, in replacing the Jβ1.1 12RSS nonamer or spacer motifs by their corresponding elements from the VκL8 12RSS (12N and 12S RSS chimeras), we verified that the increase in cleavage efficiency of 3'Dβ1-VκL8 substrate (compared to the 3'Dβ1-Jβ1.1 substrate) depended on the nonamer and spacer sequences (gels 22, 30 and 31 versus gel 13). In parallel, we did not detect any cleavage of Jκ1-Jβ1 substrate, this is probably linked to the suboptimal spacer sequence of Jκ1 23RSS, as also inferred from the similar defect displayed by the 23S chimera (gels 26, 27, 32 and 33). However, when associated to the Jβ2.4- or Jβ2.5 12RSS, the Jκ1 23- or 23S RSS had a less deleterious effect on coupled cleavage (gels 28, 29, 34 and 35) suggesting that pairing specificity is less stringent for the Jβ2 RSSs compared to Jβ1 RSSs.
