# Supplementary material for: Initiation of V(D)J Recombination by Dβ-Associated Recombination Signal Sequences: A Critical Control Point in TCRβ Gene Assembly
Source: PLoS One. 2009 Feb 24;4(2):e4575. doi: 10.1371/journal.pone.0004575 (PMC2642999; doi:10.1371/journal.pone.0004575)
Supplement: Figure S3 — RAG1/2-mediated coupled cleavage of pTCRβWT and pTCRβDMF substrates; (0.12 MB PDF) [file pone.0004575.s003.pdf]

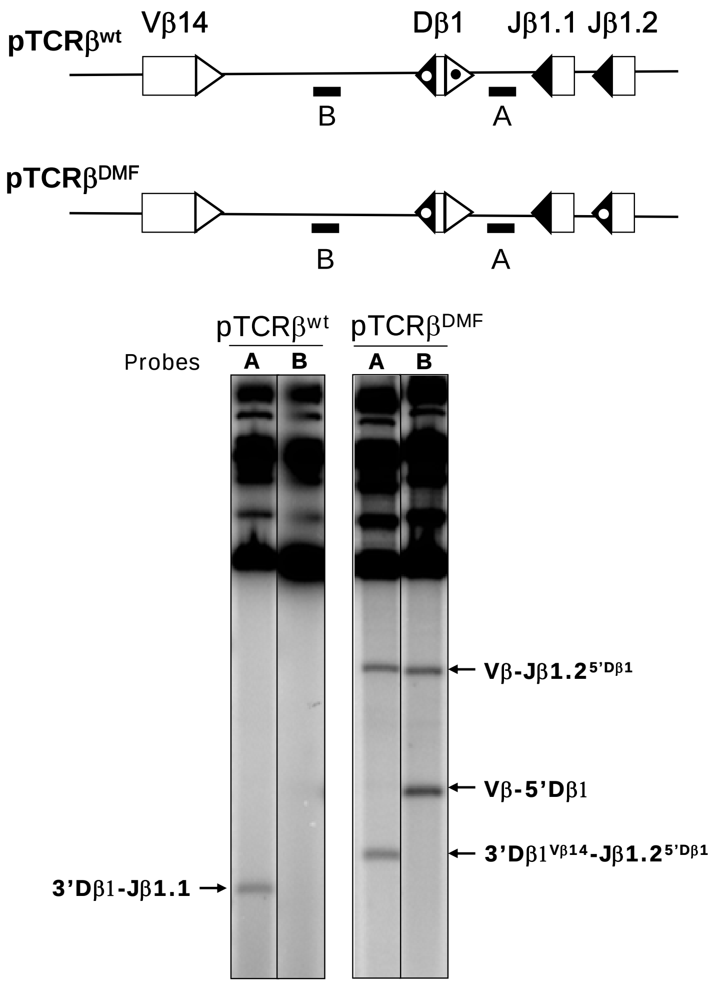

Figure S3: RAG1/2-mediated coupled cleavage of pTCRβ^{wt} and pTCRβ^{DMF} substrates.

12- and 23-RSS are respectively represented by black and white triangles. Dotted triangles correspond to Dβ1 12- and 23-RSS. In the pTCRβ^{DMF} the 3'Dβ1 23RSS is replaced by the Vβ14 23RSS (3'Dβ1^{Vβ14}) and the Jβ1.2 12RSS is replaced by the 5'Dβ1 12RSS (Jβ1.2$^{5'Dβ1}$). The localization of probes A and B used to detect signal ends products are indicated. The coupled cleavage assays were performed as described in Fig. 5B and in the Materials and Methods section. For pTCRβ^{wt} the 3'Dβ1-Jβ1.1 is detected. For pTCRβ^{DMF}, the products of the various possible coupled cleavages are detected: Vβ-Jβ1.2$^{5'Dβ1}$, the Vβ-5'Dβ1 and the 3'Dβ1^{Vβ14}-Jβ1.2$^{5'Dβ1}$. The 3'Dβ1^{Vβ14}- Jβ1.2$^{5'Dβ1}$ coupled cleavage is at least as efficient than the 3'Dβ1-Jβ1.1 coupled cleavage of the pTCRβ^{wt} substrate and therefore does not appear to be considerably slowed down. In pTCRβ^{DMF} the Vβ-5'Dβ1 coupled cleavage is not impeded by the flanking Vβ14 23RSS and is in fact quite efficient, this is consistent with our *in vivo* results indicating that at TCRβ^{DMF} minilocus some V-D rearrangements are detected before D-J rearrangements (Figure 6).
