# Supplementary material for: Initiation of V(D)J Recombination by Dβ-Associated Recombination Signal Sequences: A Critical Control Point in TCRβ Gene Assembly
Source: PLoS One. 2009 Feb 24;4(2):e4575. doi: 10.1371/journal.pone.0004575 (PMC2642999; doi:10.1371/journal.pone.0004575)
Supplement: Figure S4 — Phylogenetic profiles of Dβ and Jβ RSSs (0.15 MB PDF) [file pone.0004575.s004.pdf]

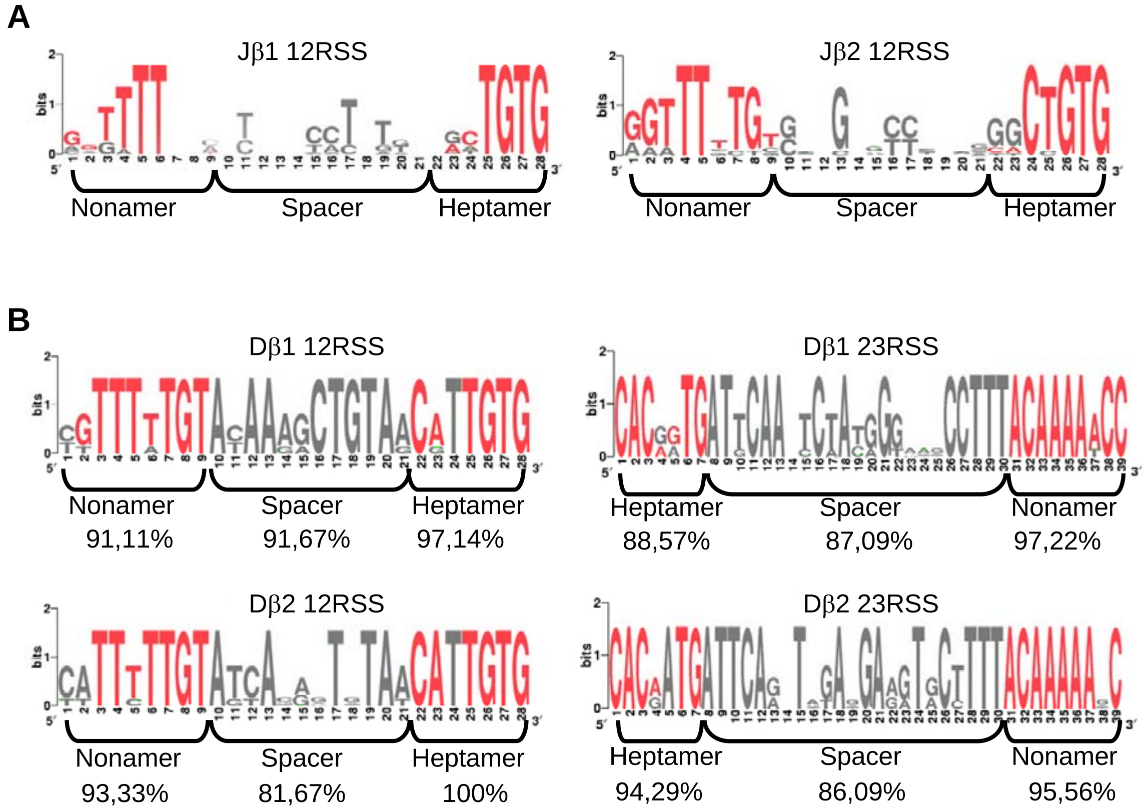

Figure S4. Phylogenetic profiles of Dβ and Jβ RSSs. (**A**) Comparative analysis of 12RSSs from the human and mouse Jβ1 (left) and Jβ2 (right) clusters. RSSs are depicted as sequence logos. PWM are represented at individual nt position by the letter's height. (**B**) Cross-species comparison between human, mouse, cow and opossum Dβ1 (top) and Dβ2 (bottom) 12- and 23RSSs. The conservation rate of the separate RSS elements across the analyzed species is indicated for each type of RSSs. (**A** and **B**) Heptamer and nonamer nt matching those in the consensus motifs [d(CACAGTG) and d(ACAAAAACC)] are in red.

All functional human and mouse Jβ and Dβ RSSs were extracted from the IMGT database (http://imgt.cines.fr/). The cow and opossum Dβ RSSs were recovered in four steps: (1) the TCRβ locus in these two species was localized by Blast using three representative mouse Vβ gene segments and the mouse Cβ regions to query the cow and opossum ensembl databases (http://www.ensembl.org); (2) the localization of TCRβ orthologues was confirmed using Blastz (http://mulan.dcode.org) to match these putative Vβ- and Cβ-contiguous loci (*i.e.*, within the sequences comprised between +700kb to -150kb upstream and downstream of the

cow and opossum Cβ regions) to those in the mouse TCRβ sequences; (3) the Dβ1 and Dβ2 gene segments were defined within these regions using Blast2seq software (http://bioweb.pasteur.fr/seqanal/interfaces/bl2seq.html) and the mouse homologous sequences as a reference; (4) the mouse, cow and opossum sequences were re-aligned using ClustalW (http://www.ebi.ac.uk/clustalw/) in order to eventually annotate the Dβ segments and cognate RSSs. The Jβ conservations were computed over all human and mouse Jβ segments. Therefore, Dβ and Jβ conservations are not comparable because of differences in methodology. Graphic representations of nucleotide conservation used Weblogo software (http://weblogo.berkeley.edu).
