# Supplementary material for: Initiation of V(D)J Recombination by Dβ-Associated Recombination Signal Sequences: A Critical Control Point in TCRβ Gene Assembly
Source: PLoS One. 2009 Feb 24;4(2):e4575. doi: 10.1371/journal.pone.0004575 (PMC2642999; doi:10.1371/journal.pone.0004575)
Supplement: Figure S5 — Effect of Dβ1 23RSS spacer mutations on RAG1/2-mediated cleavages (0.16 MB PDF) [file pone.0004575.s005.pdf]

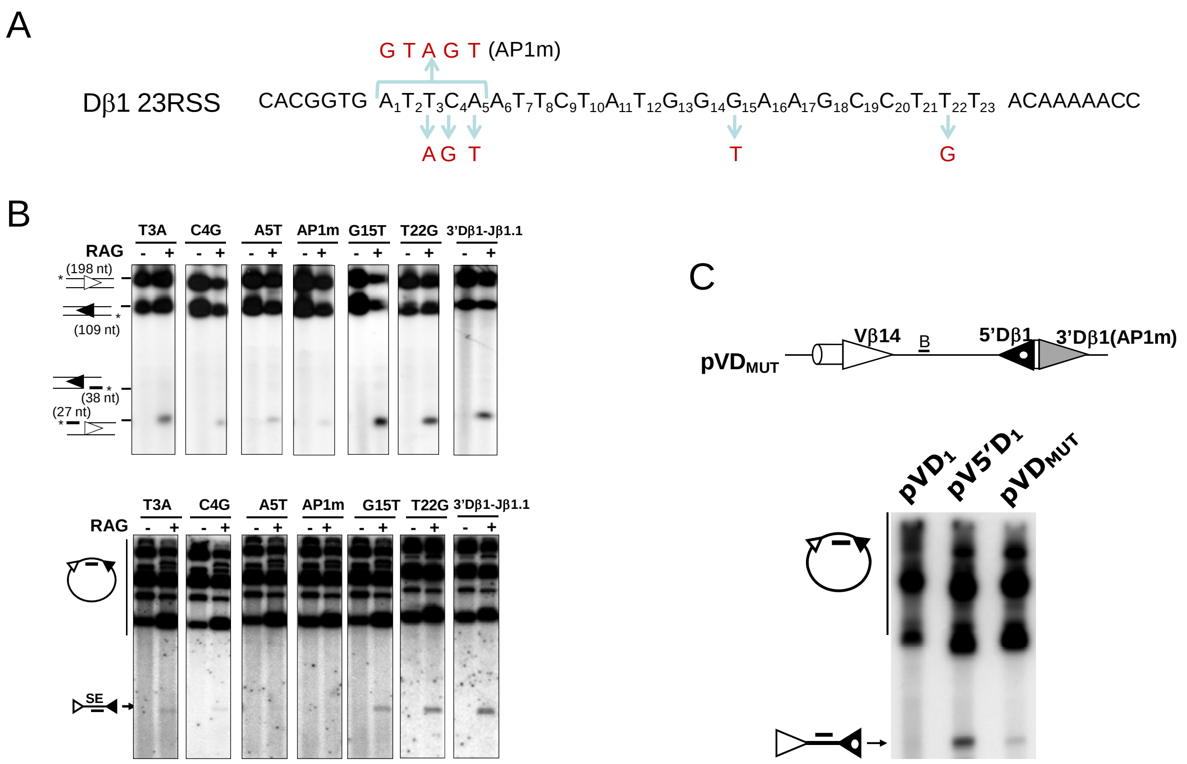


**Figure S5**: Effect of Dβ1 23RSS spacer mutations on RAG1/2-mediated cleavages.

(**A**) The T3A; C4G; A5T; G15T; T22G and AP1m mutations are depicted. In AP1m the first nucleotides of the spacer d(ATTCA) were replaced by d(GTAGT). (**B**) Within the 3'Dβ1-Jβ1.1 substrate, the 3'Dβ1 23RSS was substituted by the various 3'Dβ1 23RSS mutated versions, then the resulting substrates were named according to the mutations and were used for nick (upper panel) or coupled cleavage (lower panel) assays. Those assays were performed as described in legends of Figure 3 and Figure S2 and in material and methods section. As for the wild-type 3'Dβ1-Jβ1.1 substrate, for all the various mutated substrates, nicks were detected only at the 23RSS (27 nt fragment). However for C4G, A5T and AP1m substrates both the nicking and coupled cleavage reactions were reduced compared to wild type or the other mutated substrates. (**C**) The 3'Dβ1 23RSS within $pVD_1$ substrate was replaced by the 3'Dβ1 23RSS carrying the AP1m mutation resulting in $pVD_{MUT}$ substrate. This substrate was used in coupled cleavage assay as depicted in Figure 5B. With $pVD_{MUT}$ the amount of the coupled-cleavage product, Vβ15-5'Dβ1 SE, is higher and lower than with $pVD_1$ and $pV5'D_1$ respectively.
