# Supplementary material for: Initiation of V(D)J Recombination by Dβ-Associated Recombination Signal Sequences: A Critical Control Point in TCRβ Gene Assembly
Source: PLoS One. 2009 Feb 24;4(2):e4575. doi: 10.1371/journal.pone.0004575 (PMC2642999; doi:10.1371/journal.pone.0004575)
Supplement: Figure S6 — Cloning strategy to construct recombination substrates (0.02 MB PDF) [file pone.0004575.s006.pdf]

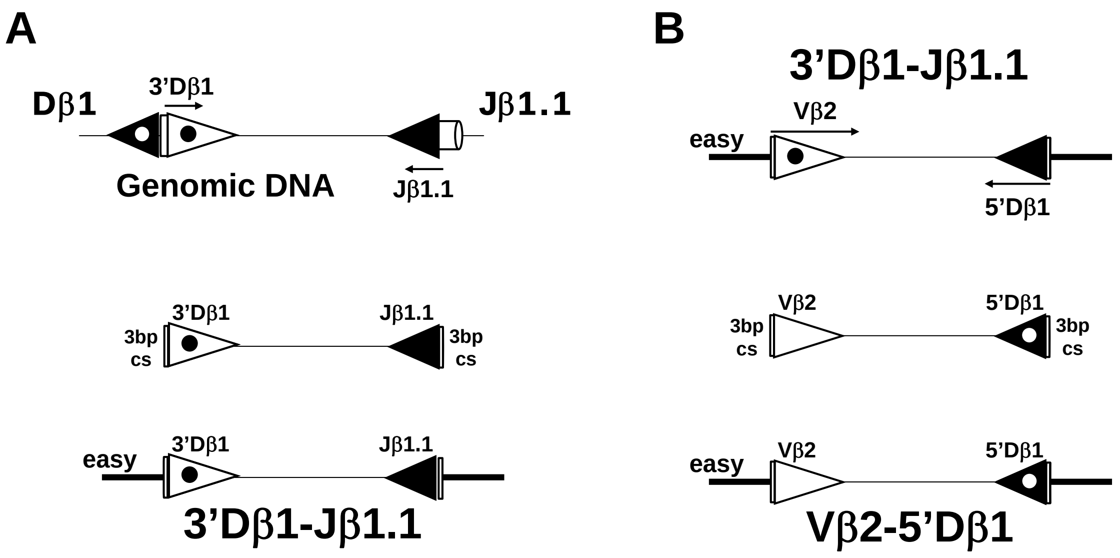

Figure S6. Cloning strategy to construct the following recombination substrates: [3'Dβ1-Jβ1.1; 3'Dβ1-Jβ1.4; 3'Dβ1-Jβ2.4; 3'Dβ1-Jβ2.5; 3'Dβ2-Jβ2.4; 3'Dβ2-Jβ2.5; Vβ2-5'Dβ1; Vβ2-5'Dβ2; Vβ2-Jβ1.1; Vβ2-Jβ1.4; Vβ2-Jβ2.4; Vβ2-Jβ2.5; Vβ14-5'Dβ1; Vβ14-5'Dβ2; Vβ14-Jβ1.1; Vβ14-Jβ1.4; Vβ14-Jβ2.4; Vβ14-Jβ2.5; Jκ1-VκL8; Vβ2-VκL8; Vβ14-VκL8; Jκ1-5'Dβ1; Jk1-5'Dβ2; 3'Dβ1-VkL8; 3'Dβ1-12N; 3'Dβ1-12S; 3'Dβ2-VκL8; Jκ1-Jβ1.1; Jκ1-Jβ1.4; Jκ1-Jβ2.4; Jκ1-Jβ2.5; 23S-Jβ1.1; 23S-Jβ1.4; 23S-Jβ2.4; 23S-Jβ2.5]. (**A**) The genomic region from the 3'Dβ1 23RSS to the Jβ1.1 12RSS (including the three proximal nucleotides from coding sequences) was amplified by PCR and subcloned into the pGEMT-easy vector, thus generating the 3'Dβ1-Jβ1.1 substrate. (**B**) All the substrates mentioned above were derived from the 3'Dβ1-Jβ1.1 substrate, using PCR amplification and oligonucleotide primers listed in **Table S4**. For example, using the 3'Dβ1-Jβ1.1 substrate as a template along with the Vβ2 and 5'Dβ1 primers, we produced the Vβ2-5'Dβ1 fragment. The latter fragment was then subcloned into pGEMT-easy to generate the Vβ2-5'Dβ1 substrate. Hence, all the substrates possess the same backbone, except that they differ in their RSSs and 3 bp of flanking (coding) sequences.
