# Supplementary material for: Initiation of V(D)J Recombination by Dβ-Associated Recombination Signal Sequences: A Critical Control Point in TCRβ Gene Assembly
Source: PLoS One. 2009 Feb 24;4(2):e4575. doi: 10.1371/journal.pone.0004575 (PMC2642999; doi:10.1371/journal.pone.0004575)
Supplement: Figure S7 — Cloning strategy to construct additional recombination substrates (0.03 MB PDF) [file pone.0004575.s007.pdf]

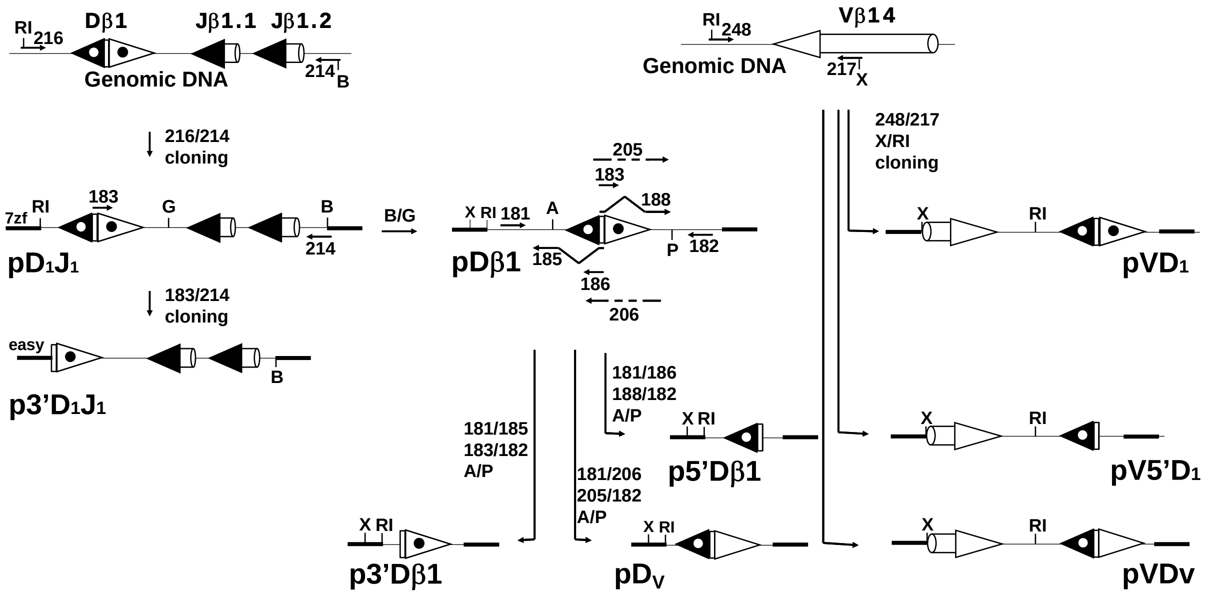

Figure S7. Cloning strategy to construct additional recombination substrates: [pD$_1$J$_1$; p3'D$_1$J$_1$; pDβ1; p5'Dβ1; pDv; p3'Dβ1; pVD$_1$; pV5'D$_1$; pVDv]. Genomic DNA encompassing the Dβ1 to Jβ1.2 region was amplified by PCR using primers #216 and #214 (see the primer list in **Table S4**) and subcloned into pGEM-7f using *Eco*RI and *Bam*HI sites, thus yielding substrate pD$_1$J$_1$. The PCR product resulting from amplification with primers #183 and #214 was subcloned into pGEMT-easy yielding substrate p3'D$_1$J$_1$. The *Bgl*II/*Bam*HI fragment excised from pD$_1$J$_1$ yielded the pDβ1 template. The latter template was mutated in a 2-steps PCR process: (*i*) 1st amplification used primers #181 and #186 or #188 and #182; (*ii*) 2nd amplification (from the resulting PCR product) used most 5' and 3' primers (#181 and #182). The latter product was digested and re-inserted into the *Acc*I/*Pst*I sites of the same vector to produce the p5'Dβ1 substrate. A similar strategy was applied to generate constructs pDv and p3'Dβ1 [PCR amplifications then used primers pairs #181 and #206 followed by #205 and #182 (pDv); or #181 and #185 followed by #183 and #182 (p3'Dβ1)]. A genomic fragment containing the Vβ14 23RSS was PCR amplified using primers #248 and #217. The resulting product was digested with *Eco*RI and *Xba*I and subcloned into pDβ1, p5'Dβ1 or pDv, thus yielding substrates pVD$_1$, pV5'D$_1$ or pVDv, respectively. RI, *Eco*RI; G, *Bgl*II; B, *Bam*HI; S, *Sac*I; X, *Xba*I; A, *Acc*I; P, *Pst*I.
