# Supplementary material for: Initiation of V(D)J Recombination by Dβ-Associated Recombination Signal Sequences: A Critical Control Point in TCRβ Gene Assembly
Source: PLoS One. 2009 Feb 24;4(2):e4575. doi: 10.1371/journal.pone.0004575 (PMC2642999; doi:10.1371/journal.pone.0004575)
Supplement: Table S1 — 5′-phosphorylated 3′-biotinylated oligonucleotides (7-mers) and restriction enzymes used in the oligo-capture assays to displace and ligate the nicked strand and to restrict the genomic DNA before purification on streptavidin-conjugated magnetic beads. (0.05 MB PDF) [file pone.0004575.s008.pdf]

| RSS of gene segments targeted | Heptamer (5'→3') | Restriction enzymes used for digestion |
|---|---|---|
| **Vβ14** | **p-CACACTG-Biotin** | ***Bgl*II; *Eco*RI; *Hind*III; *Pvu*II; *Sph*I; *Xba*I** |
| **Vβ2; Vβ6; Vβ15; Vβ8** | **p-CACAGTG-Biotin** | ***Bgl*II; *Hind*III; *Sst*I; *Sph*I; *Xba*I** |
| **Vβ4(Vβ16); Vβ5** | **p-CACAGCC-Biotin** | ***Bgl*II; *Eco*RI *Hind*III; *Nco*I; *Sph*I** |
| **5'Dβ1<br>Dβ2** | **p-CACAATG-Biotin** | ***Bgl*II; *Eco*RI; *Eco*RV; *Hind*III; *Pvu*II; *Sph*I; *Sst*I; *Xba*I** |
| **3'Dβ1** | **p-CACGGTG-Biotin** | ***Bgl*II; *Eco*RI; *Eco*RV; *Hind*III; *Pvu*II; *Sph*I; *Sst*I; *Xba*I** |
| **Jβ1.1<br>Jβ1.2<br>Jβ1.3<br>Jβ1.4<br>Jβ1.5<br>Jβ1.6** | **p-CACAGTG-Biotin<br>p-CACATCA-Biotin<br>p-CACAGCC-Biotin<br>p-CACAACA-Biotin<br>p-CACAGTA-Biotin<br>p-CACAGCT-Biotin** | ***Bgl*II; *Eco*RI; *Hind*III; *Sst*I; *Xba*I** |
| **Jβ2.1<br>Jβ2.2<br>Jβ2.3<br>Jβ2.4<br>Jβ2.5<br>Jβ2.7** | **p-CACAGCA-Biotin<br>p-CACAGTC-Biotin<br>p-CACAGCC-Biotin<br>p-CACAGCC-Biotin<br>p-CACAGCC-Biotin<br>p-CACAGAG-Biotin** | ***Bgl*II; *Eco*RI; *Eco*RV; *Hind*III; *Sph*I; *Xba*I** |

**Table S1.** 5'-phosphorylated 3'-biotinylated oligonucleotides (7-mers) and restriction enzymes used in the oligo-capture assays to displace and ligate the nicked strand and to restrict the genomic DNA before purification on streptavidin-conjugated magnetic beads.
