# Supplementary material for: Initiation of V(D)J Recombination by Dβ-Associated Recombination Signal Sequences: A Critical Control Point in TCRβ Gene Assembly
Source: PLoS One. 2009 Feb 24;4(2):e4575. doi: 10.1371/journal.pone.0004575 (PMC2642999; doi:10.1371/journal.pone.0004575)
Supplement: Table S2 — DNA sequences of the RSSs (plus the three proximal nucleotides from coding flanks) used in this study. (0.01 MB PDF) [file pone.0004575.s009.pdf]

Gene segment	Coding sequence	heptamer	spacer	nonamer
Jκ1[b]	GGC	CACAGTG	GTAGTACTCCACTGTCTGGCTGT	ACAAAAACC
3′Dβ1	GGC	CACGGTG	ATTCAATTCTATGGGAAGCCTTT	ACAAAAACC
3′Dβ2	GGC	CACAATG	ATTCAACTGGAAGAGGTGCTTTT	ACAAAAAGC
Vβ2	AGA	CACAGTG	GTAAACTCTGCAGGCGCATTGAA	ACAAAAACC
Vβ14	TCT	CACACTG	AGTAGGGTGGGGCAGACATCTGT	GCAAAAACC
23S[a,b]	GGC	CACGGTG	GTAGTACTCCACTGTCTGGCTGT	ACAAAAACC
VκL8[b,c]	TTG	CACAGTG	CTACAGACTGGA	ACAAAAACC
5′Dβ1	CCC	CACAATG	TTACAGCTTTAT	ACAAAAAAG
5′Dβ2	CCC	CACAATG	TTACATCGTGAT	ACAAAAAAG
Jβ1.1	TTG	CACAGTG	CCATAGGATGAG	GAGAAAAAT
Jβ1.4	AAA	CACAACA	TTAAAGCCTGGT	GGTAAAACT
Jβ2.4	ACT	CACAGCC	TCTTGGTACAGG	ACAAAAACT
Jβ2.5	GTT	CACAGCC	CCAGAACCCAAC	ACAAAAACT
12S[a,b]	TTG	CACAGTG	CTACAGACTGGA	GAGAAAAAT
12N[a,b]	TTG	CACAGTG	CCATAGGATGAG	ACAAAAACC

Table S2. DNA sequences of the RSSs (plus the three proximal nucleotides from coding flanks) used in this study. The RSSs are named according to their corresponding gene segment. [a]RSS chimeras of TCRβ- and IgLκ-derived sequences. [b]12- and 23RSSs that are bordered by 3 nucleotides of coding flanks from the Jβ1.1 and 3’Dβ1 gene segments, respectively. [c]The nonamer sequence of the VκL8 12RSS has been modified to match the corresponding consensus motif.
