# Supplementary material for: Initiation of V(D)J Recombination by Dβ-Associated Recombination Signal Sequences: A Critical Control Point in TCRβ Gene Assembly
Source: PLoS One. 2009 Feb 24;4(2):e4575. doi: 10.1371/journal.pone.0004575 (PMC2642999; doi:10.1371/journal.pone.0004575)
Supplement: Table S3 — Oligonucleotide primers and hybridization probes used in the oligo-capture assays for PCR amplification and Southern blotting identification of the captured DNAs (0.05 MB PDF) [file pone.0004575.s010.pdf]

Gene segment targeted	Forward (5'→3')	Probe (5'→3')	Reverse (5'→3')
Vβ14	GCACAGAGGTAGAAGCCAGAGTGG	AGAGTCGGTGGTGCAACTGAACCT	GATTACATGTAACCCCTTCAGAGGG
Vβ2	TCCTTTCGTCTTGTTTCAGACCCC	TGTGGCCAGGTAATCAGCACC	TGCAGGTGCAGTACAAGGTTCTGC
Vβ6	TGTTGCCGAGTGCACATCTTAACCC	AAGGCGATCTATCTGAAGGCTATGA	AAACGGCCATCTCGTTCTTCTGGG
Vβ15	GGAGTGAACCCAAATTTTCTGGG	TGAGGATGGAGTGTCAAGCTG	GGATAAGTTGGGATGACTGATGGG
Vβ8	GTGAYRTCACTAAGTCACTGAAAGMCCC*	AACACATGGAGGCTGCAGTCACC	TAATGGATCAGCCTCAGCCCATGC
Vβ4(Vβ16)	TGAGTCCTAGTCTTGGCCTATACACC	TATCTGGTGGCAGTCACAGGGA	TTGGCTGCTGGCACAGAAGTATGTGG
Vβ5	TGTCTCCTTCCTCCCCAGGTTCAGC	CCCAGCAGATTCTCAGTCCAACAG	TGGCACAGAAGTACAYRGCAGAGTCC*
5'Dβ1	GATTGGGGGCTGTTACTTCTTCATAGGG	CAATGACACCCAGCGC	TTGTGCAAGGTGGTGGTAAGATGC
3'Dβ1	ATTCTGTCTGTCCCAAGGCCC	GCTGCTAGGGCCACTAGGC	TGTAGAACTGTTCACCTCTGGC
Jβ1	TAATTCGCCCCTCTACTTTGCGGC	CTCTACTTTGCGGCAGGCACC	CAGAAGTCACTGAAGACAGTAGCC
Dβ2	TCCCACCCATTTCTCTATAAATGCC	TACCCAGCTTGAGACTTTTCCCAGC	TTTCTTCCCCACAGGTGCCTACCC
Jβ2	TATGAACAGTACTTCGGTCCC	TGAGAGCTGTCTCCTACTATCGATT	TTGTCCTGGCTTGCGAGAGAGC
Cβ2	TGTGGCAGGCTCTAATTAAAT	ATTCACCCACCAGCTCAGCTCCACGTG	GCTATAATTGCTCTCCTTGATGGCCTG

Table S3. Oligonucleotide primers and hybridization probes used in the oligo-capture assays for PCR amplification and Southern blotting identification of the captured DNAs. *: Primers that contain moderate degeneracy [Y (= C or T), R (= A or G), M (= A or C)] in order to amplify all Vβ8 (Vβ8.1, Vβ8.2 and Vβ8.3) or Vβ5 (Vβ5.1 and Vβ5.2) gene segments.
