# Supplementary material for: Initiation of V(D)J Recombination by Dβ-Associated Recombination Signal Sequences: A Critical Control Point in TCRβ Gene Assembly
Source: PLoS One. 2009 Feb 24;4(2):e4575. doi: 10.1371/journal.pone.0004575 (PMC2642999; doi:10.1371/journal.pone.0004575)
Supplement: Table S4 — Oligonucleotides used in the construction of the various DNA cleavage substrates analyzed in this study. (0.03 MB PDF) [file pone.0004575.s011.pdf]

Name	Sequence (5’→3’)
Jκ1	GGCCACAGTGGTAGTACTCCACTGTCTGGCTGTACAAAAACCATTCTGTCTG
3’Dβ1	GGCCACGGTGATTCAATTCTATG
3’Dβ2	GGCCACAATGATTCAACTGGAAGAGGTGCTTTTACAAAAAGCTCTCTGTCTGTCCCAAGGC
Vβ2	AGACACAGTGGTAAACTCTGCAGGCGCATTGAAACAAAAACCACACTGTCTGTCCCAAGGC
Vβ14	TCTCACACTGAGTAGGGTGGGGCAGACATCTGTGCAAAAACCACACTGTCTGTCCCAAGGC
23S	GGCCACGGTGGTAGTACTCCACTGTCTGGCTGTACAAAAACCATTCTGTCTG
VκL8	TTGCACAGTGCTACAGACTGGAACAAAAACCAATCTTAAGGGG
5’Dβ1	CCCCACAATGTTACAGCTTTATACAAAAAAGGACCTTAAGGGGTGAAGAGAGGC
5’Dβ2	CCCCACAATGTTACATCGTGATACAAAAAAGTTTCTTAAGGGGTGAAGAGAGGC
Jβ1.1	TTGCACAGTGCCATAGGATGAGG
Jβ1.4	AAACACAACATTAAAGCCTGGTGGTAAAACTAATCTTAAGGGG
Jβ2.4	ACTCACAGCCTCTTGGTACAGGACAAAAACTGGCCTTAAGGGGTGAAGAGAGGC
Jβ2.5	GTTCACAGCCCCAGAACCCAACACAAAAACTATACTTAAGGGGTGAAGAGAGGC
12S	TTGCACAGTGCTACAGACTGGAGAGAAAAATAATCTTAAGGGG
12N	TTGCACAGTGCCATAGGATGAGACAAAAACCATTCTTAAGGGGTGAAGAGA
181	CAATGACACCCAGCGC
182	GCTGCTAGGGCCACTAGGC
183	GGGACAGGGGGCCACG
185	GCCCCCTGTCCCGACCCTCCCATAGG
186	GCCCCCTGTCCCCACAATG
188	GGGACAGGGGGCATTCTGTCTGTCCC
205	GGGGGCCACACTGAGTAGGGTGGGGCAGACATCTGTGCAAAAACCATTCTG
206	GAATGGTTTTTGCACAGATGTCTGCCCCACCCTACTCAGTGTGGCCCCCTGTCC
207	CCGTGTCCTTTTTTGTATAAAGCTGTAACATTGTGCAAACTCCG
208	CGGAGTTTGCACAATGTTACAGCTTTATACAAAAAAGGACACGGAGGAC
213	CAGTGGATAGGTGAGCCAGAGG
214	TTGTGGATCCAGCCTCATTAGAATGTAGTCC
216	ATGTGGGAATTCATTTCAATG ACACCCAGCG
217	TGCTCCTCTAGAGTCGGTGGTGCAACTGAACC
248	GGAGTTGAATTCCTCTTCTTCATTTCCATCC
318	CCATTCTTCTAGACAATCTTGGCCTAGCAGGC

Table S4. Oligonucleotides used in the construction of the various DNA cleavage substrates analyzed in this study.
