# Supplementary material for: Initiation of V(D)J Recombination by Dβ-Associated Recombination Signal Sequences: A Critical Control Point in TCRβ Gene Assembly
Source: PLoS One. 2009 Feb 24;4(2):e4575. doi: 10.1371/journal.pone.0004575 (PMC2642999; doi:10.1371/journal.pone.0004575)
Supplement: Text S1 — Consideration of an Alternative capture model. (0.07 MB PDF) [file pone.0004575.s012.pdf]

Text S1. **Consideration of an Alternative capture model.**

The basis of the capture model is that the RAG1/2 multimers complex assembles on one RSS (or nucleating RSS) and then captures the RAG-free RSS partner (or captured RSS) [1,2]. Consequently RAG1/2 binding to the nucleating RSS is independent of the synapse. Moreover it has been shown that the nicking step *per se* is an unireactant reaction independent of the synapse [3], therefore the amount of nicked nucleating RSS should be constant (wherever the nicking is occurring in or out of the synapse). The capture model also implies that RAG1/2 binding to the captured RSS is dependant of the synapse, and thus that the nicking of the captured RSS occurs only in the synapse.

To depict the capture model we took into consideration the analysis and interpretation by Curry et al. of nicked RSS profiles at Igk, IgH and TCRα loci [4].Therefore in the capture model described in **Figure 1A**, the first nick occurs at the nucleating RSS and is independent of the synapse whereas the 2nd nick occurs at the captured RSS within the synapse.

Here we considered an alternative capture model (schematized below) in which the first nick occurs at the captured RSS (white triangle) within the synapse. Then, the 2nd nick occurs at the nucleating RSS (black triangle). Thus, the initiating RSS is bound by RAG1/2 in step 1 and is nicked only at step 4. This implies that the kinetic of the nicking step of the nucleating RSS is considerably slow.

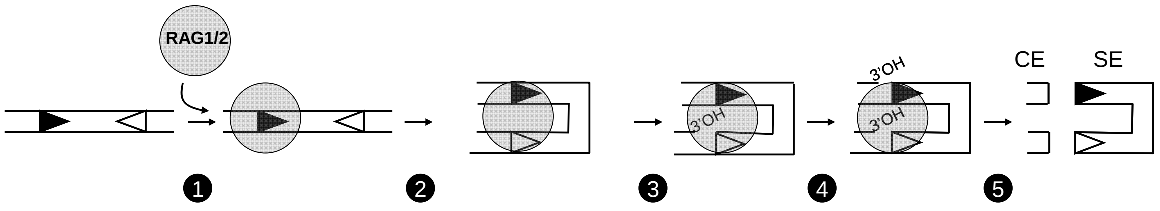

For the various potential configurations, we challenged this alternative model with our data.

1°) Dβ 23RSS nucleates synapsis and captures Jβ RSS. Then the first nick occurs within the synapse at Jβ RSS. This is not consistent with both *in vitro* and *in vivo* data because Jβ RSS nicks are not detected whereas Dβ 23RSS nicks are readily detected (Figure 2C and Figure 3 gels 9 and 10). Therefore according to this alternative model Jβ RSS cannot be the captured RSS.

2°) <u>Jβ RSS nucleates synapsis and captures Dβ 23RSS</u>. Then the first nick occurs within the synapse at Dβ 23RSS. This is not consistent with *in vitro* data because the formation of nicked Dβ1 23RSS is independent of capture/synapsis (i.e. it efficiently occurs on isolated Dβ1 23RSS) (depicted on the right, also shown in Figure 5A gel 2 and Figure 3 gel 9). Therefore the Dβ1 23RSS cannot be the captured RSS.

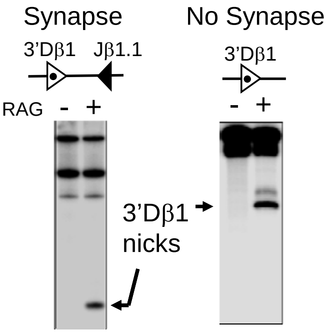

Nicking at 3'Dβ1 23RSS
is independant of synapsis

3°) <u>Dβ 12RSS nucleates synapsis and captures Vβ RSS</u>. Then the first nick occurs within the synapse at Vβ RSS. This is not consistent with both *in vitro* and *in vivo* data because (i) the kinetics of Dβ 12RSS nicking is fast and (ii) Dβ 12RSS nicks (but not Vβ 23RSS nicks) are detected *in vivo* (see Figure 2C and Figure 3 gels 5 to 8). Therefore Vβ RSS can not be the captured RSS.

4°) <u>Vβ RSS nucleates synapsis and captures Dβ 12RSS.</u> Then the first nick occurs within the synapse at Dβ 12RSS. This is not consistent with *in vitro* data because (i) formation of Vβ RSS nicks are stimulated by synapsis (one example is reported below on the left; also shown in Figure 3 compare gels 1-4 to gels 5-8); and (ii) formation of nicked Dβ1 12RSS is independent of synapsis (see below right; also shown in Figure 5A gel 1 and Figure 3 gels 5-6). Therefore the Dβ1 12RSS cannot be the captured RSS.

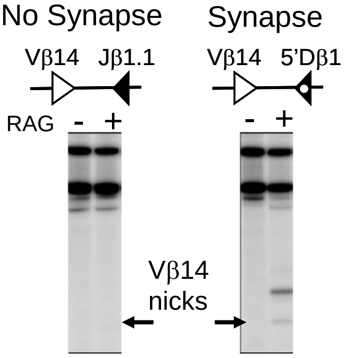

Nicking at Vβ 23RSS is not constant:
No Vβ nick for Vβ-Jβ substrates whereas
Vβ nicks are readely detected for Vβ-5'Dβ subtrates

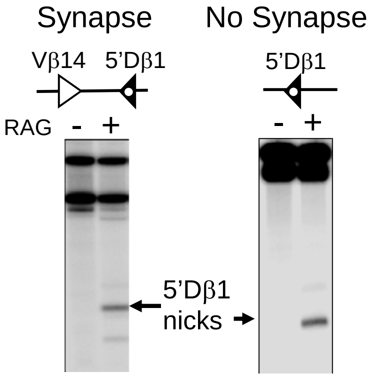

Nicking at 5'Dβ1 12RSS
is independant of synapsis

In conclusion, when considering any of the various possibilities for D-J and V-D TCRβ RSS pairs, our data do not match the alternative model. By contrast, our results agree with and strengthen Schlissel's interpretation: the first nick occurs at the initiating RSS (but not at the captured RSS) likely before synapsis [4].

As proposed in the discussion and depicted below, we suggested that in step 1 the RAG1/2 "free" conformation (dark grey) would not allow efficient binding or nicking reaction of the captured RSS (then the RAG:RSS complex may dissociate, releasing an intact RSS). In the other hand this "free" form of RAG1/2 binds and nicks the nucleating RSS. In this single complex the structure of the RAG1/2 multimers changes and then conversely to the "free" conformation, this new "complexed" conformation of RAG1/2 (light grey), would allow the efficient binding (or the subsequent nicking step) of the captured RSS.

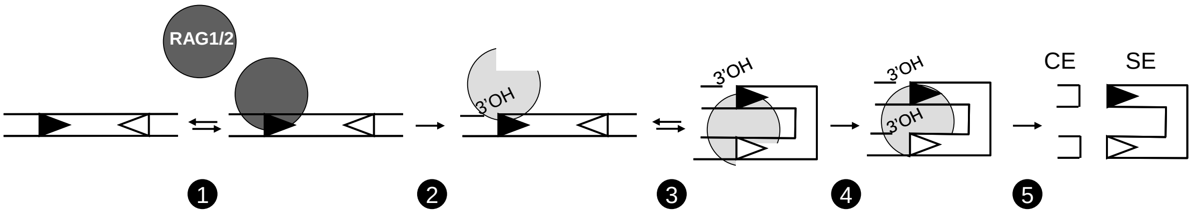

References

1. Jones JM, Gellert M (2002) Ordered assembly of the V(D)J synaptic complex ensures accurate recombination. EMBO J 21: 4162-4171.

2. Mundy CL, Patenge N, Matthews AG, Oettinger MA (2002) Assembly of the RAG1/RAG2 synaptic complex. Mol Cell Biol 22: 69-77.

3. Yu K, Lieber MR (2000) The nicking step in V(D)J recombination is independent of synapsis: implications for the immune repertoire. Mol Cell Biol 20: 7914-7921.

4. Curry JD, Geier JK, Schlissel MS (2005) Single-strand recombination signal sequence nicks in vivo: evidence for a capture model of synapsis. Nat Immunol 6: 1272-1279.
